# Full-length direct RNA sequencing reveals widespread RNA decay upon cellular stress

**DOI:** 10.1101/2023.08.31.555629

**Published:** 2023-09-01

**Authors:** Showkat A. Dar, Sulochan Malla, Christopher T. Lee, Matthew J. Payea, Jessica Martin, Aditya J. Khandeshi, Jennifer L. Martindale, Cedric Belair, Manolis Maragkakis

**Affiliations:** Laboratory of Genetics and Genomics, National Institute on Aging, Intramural Research Program, National Institutes of Health, Baltimore, MD 21224, USA

## Abstract

Cells respond and adapt to endogenous and exogenous stressors by activating stress response pathways that restore cellular homeostasis. The capacity of cells to respond to stress declines with age and has been associated with disease. However, the changes in cytoplasmic RNA metabolism during the cellular stress response are poorly characterized. In this work, we employ end-to-end direct RNA sequencing with nanopores, following the ligation of unique 5’ end adaptors, to assess the human transcriptome at single-molecule and -nucleotide resolution. To analyze this data, we developed NanopLen, a statistical tool to identify robust RNA length changes across conditions using linear mixed models. We find that upon cellular stress, RNAs are globally, subject to decay at their 5’ end. Stress-induced decay is coupled to translation and ribosome occupancy and can be inhibited by restoring translation initiation or preventing ribosome run-off. In contrast to models of RNA decay under normal conditions, we show that stress-induced RNA decay is dependent on XRN1 but is independent of the poly(A) tail length. Surprisingly, we find that decaying RNAs persist in cells and are enriched in the stress granule transcriptome. Our results reveal the dynamics of post-transcriptional cell response that regulates the state of RNA upon cellular stress.

## Introduction

Cells encounter and need to respond to a wide variety of exogenous and endogenous conditions that induce cellular stress. Stressed cells employ a cascade of molecular changes and activate specialized pathways that aim to restore homeostasis or induce apoptosis ^1^. The capacity of cells to activate these pathways deteriorates with age ^3^ and has been associated with many diseases, particularly age-related neurodegeneration ^2,4^. Oxidative stress (OS) caused by an imbalance between production and accumulation of reactive oxygen species (ROS) ^5^, is associated with aging and age-related disease, and has been a primary target for therapy and biomarker development ^8^. OS induces the Integrated Stress Response (ISR), a common adaptive pathway that eukaryotic cells activate to restore cellular homeostasis ^10,25^. ISR involves the phosphorylation of eukaryotic translation initiation factor 2 alpha (eIF2α) that inhibits translation initiation and decreases the global protein synthesis ^13^. In turn, mRNAs exiting the translational pool are localized in stress granules (SGs), membraneless organelles that are formed when translation of numerous RNAs is arrested ^15,17^.

Translation is intimately coupled to RNA stability and recent studies have shown that RNA can degrade co-translationally ^19,21^. Similarly, aberrant translation elongation that is induced by ribosome stalling on RNAs leads to RNA degradation via the No-Go decay (NGD) surveillance pathway ^23,26,27^. In yeast, ribosomal and other RNA binding proteins (RBPs) that contact mRNAs to facilitate translation under physiological conditions, instead show diminished mRNA association upon stress ^11^. Using short-read RNA-Seq, it has been shown that this reduction results in decreased mRNA abundance upon heat shock but not upon glucose withdrawal, mediated by the 5′–3′ exoribonuclease Xrn1 ^11^. Similar studies have also shown that Xrn1 deletion leads to accumulation of oxidized RNAs bearing nucleotide adducts such as 8-oxoguanosine (8-oxoG), leading to translation inhibition and NGD ^12^. Thus, there are at least two distinct pathways that link stress-associated translation inhibition with reduced RNA stability in yeast. However, the dynamics of the involved pathways are still largely unexplored, particularly in human cell systems.

The global effects of stress on individual RNA molecules at single nucleotide resolution have been particularly elusive, with studies done almost exclusively using artificial reporters. Also, traditional short-read RNA sequencing approaches rely on artificial fragmentation of RNA thus convolving RNA abundance with decay. In this work, we globally profile the integrity of individual RNA molecules in human cells by full-length direct RNA sequencing with nanopores (dRNA-Seq). We develop a new computational framework for statistical interrogation of RNA decay and find that upon cellular stress, RNAs exhibit marked shortening at their 5′ end that is mediated by XRN1 independently of prior de-adenylation. Treating cells to restore translation initiation or prevent ribosome run-off during stress inhibits stress-induced RNA decay associating degradation to ribosome occupancy. Importantly, by decoupling RNA decay from abundance, we reveal that the decaying RNA molecules persist in cells and are enriched in the SG transcriptome. Our results highlight the dynamics of a cellular stress response mechanism regulating the state of RNA and establish a statistical tool for the analysis of long-read direct RNA sequencing data.

## Results

### Stress induces widespread decay at the 5’ end of RNAs

**Fig 1:**
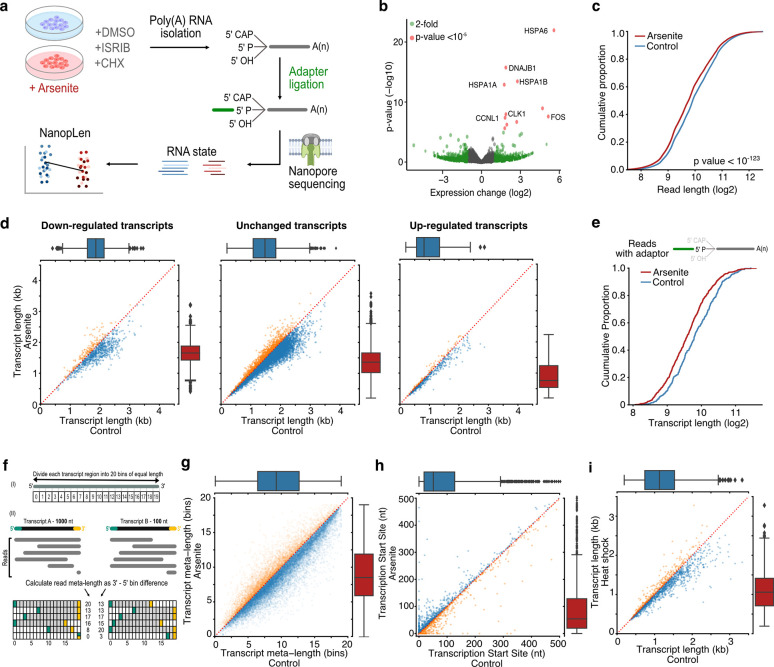
RNA shortening upon cellular stress. **a)** Schematic of experimental design **b)** Volcano plot for the differential expression of arsenite-treated and control cells. Green color indicates genes with more than 2-fold difference and red indicates statistical significance higher than 10^−5^. **c)** Cumulative distribution of read length for arsenite-treated and control cells. **d)** Scatter plots of average transcript length for arsenite-treated and control cells stratified by their differential expression change. Down-regulated: (-Inf, −0.5), Unchanged: (−0.5, 0.5), Up-regulated (0.5, Inf) fold-change. Only transcripts with at least 5 aligned reads are shown. Red dotted indicates the y=x line. Color indicates transcripts below (blue) and above (orange) the diagonal. **e)** Cumulative distribution of transcript length for arsenite-treated and control cells using only reads with adaptor ligated at the 5’ end. **f)** Schematic of read meta-length calculation. Each annotated transcript is divided into 20 equally sized bins. Each read is then assigned meta-coordinates depending on the bin in which its 5’ and 3’ ends align. The read meta-length is calculated as the difference of the meta-coordinates and presented as a percentage of full length. **g)** Scatter plot of average transcript meta-length as a percentage of full-length for arsenite-treated and control cells. Coloring is the same as (d) **h)** Scatter plot of Transcription Start Site position for arsenite-treated and control cells **i)** Scatter plot of average transcript length for heat shock and control cells.

Human cells at steady-state, have been shown to harbor a surprising majority of RNA molecules that exist in a transient, partially degraded state ^21,28^. However, how this balance between intact and partially degraded RNA adjusts, and is regulated in non-steady-state conditions, such as cellular stress, remains unclear. To explore this, we first triggered OS by subjecting HeLa cells to sodium arsenite (0.5 mM) for 60 minutes. We verified the anticipated formation of SGs and phosphorylation of eIF2α (discussed in subsequent sections) while cell viability was monitored at varied intervals up to a day after treatment with no major changes observed (Sup. Fig. 1a). We subsequently collected arsenite-treated and untreated cells (control) and performed TERA-Seq ^28^ (Fig. 1a). TERA-Seq involves dRNA-Seq thus profiles the transcriptome at single molecule resolution without bias from reverse-transcription or PCR amplification ^14–16^. Additionally, it incorporates unique adapters that are ligated to the 5’ phosphorylated end of individual RNA molecules, which are then sequenced along with the native sequence. Since nanopore-based RNA sequencing proceeds in the 3’ to 5’ direction, the inclusion of an adapter at the 5’ end of a read acts as an internal control that ensures that an RNA molecule has been read in its entirety. Nevertheless, the method doesn’t explicitly select for ligated molecules, thus the resultant datasets comprise a mix of molecules, irrespective of their ligation to adapters. Ligated molecules can subsequently be computationally recognized and separated to be analyzed jointly or independently to non-ligated reads. Samples were prepared in triplicates and sequenced on a MinION device, yielding approximately 2 million long-reads per library (Sup. Table 1). Replicates displayed high expression correlation and high alignment efficiency to the human genome (Sup. Fig. 1b, Sup. Table 1). Consistent with OS induction, differential gene expression analysis identified several stress response genes being upregulated in arsenite while gene ontology (GO) enrichment analysis of differentially expressed genes showed expected associations to cellular response to stress (Fig. 1b, Sup. Fig 1c, Sup. Table 3). These results show that TERA-Seq accurately and reproducibly captures the transcriptome state upon OS.

While these data showed anticipated gene abundance changes, quantification of distribution of read lengths in the two conditions showed an unexpected and statistically significant read length difference with reads in OS being shorter by an average of 115.3 nucleotides (nts) compared to control (Mann–Whitney: p-value < 10^−123^, Fig. 1c). This analysis grouped reads from all genes together, thus could be biased by a small subset of genes or by differential expression of longer or shorter genes. Therefore, to explore this finding further, we calculated a new metric which we refer to as average transcript length which is quantified as the average length of reads aligning to each transcript (herein the term transcript is used to refer to annotated gene isoforms, not individual RNA molecules). Additionally, we stratified transcripts by their corresponding differential expression i.e., up-, down-regulated and unchanged, to explore whether the observed shortening can be attributed to expression change. Our results showed a robust and almost global RNA shortening that occurred independently of transcript expression (Fig 1d) or coding potential (Sup. Fig. 1d and 1e). To confirm that this was not due to library preparation artifacts or failure to capture long transcripts by the sequencing device, we repeated the analysis by computationally selecting only reads with ligated 5′ adaptors, ensuring RNA molecules were fully sequenced. The subset of 5′ adaptor-ligated reads confirmed our findings and again showed global RNA shortening upon OS (Fig. 1e). Since both ligated and non-ligated reads showed comparable results, in subsequent analysis we used all reads unless otherwise mentioned.

To test whether stress induced RNA shortening depends on preexisting steady-state fragmentation levels, we also performed analyses in a length-normalized space (meta-length) that represents read length as a percent of annotated transcript length. We calculated normalized meta-length by splitting each annotated transcript in 20 equal bins, assigning the mapped read ends into individual bins, and defining the transcript meta-length as the difference of the 5’ and 3’ end bins (Fig. 1f). Our data showed a consistent reduction of transcript meta-length upon OS, independent of the position on the x-axis, indicating that transcripts are being shortened independently of steady-state fragmentation levels (Fig. 1g, Sup. Fig. 1f). We also considered the possibility that RNA shortening could be attributed to selection of alternative transcription start sites (TSSs) downstream of annotated TSSs. We have previously established that, in TERA-Seq, positions with high read density upstream of coding sequences accurately represent TSSs and match those defined by CAGE-Seq ^28^. We therefore employed this approach to independently calculate TSSs in stress and control conditions. Our results showed only minor changes between conditions (Mann–Whitney: p-value = 0.35) with no preference towards shorter or longer transcripts (Fig. 1h) indicating that the observed shortening likely occurs post-transcriptionally.

To exclude potential effects of the arsenite chemical itself, we further tested OS induction with 0.3 mM hydrogen peroxide (H_2_O_2_) for 2 hours followed by TERA-Seq. H_2_O_2_-induced OS also resulted in significant transcript shortening (Mann–Whitney: p-value < 10^−96^), similar to that observed with arsenite, indicating that OS results in RNA shortening irrespective of inducer (Sup. Fig. 1g). To explore whether RNA shortening is unique to OS or constitutes a general stress response, we re-examined publicly available dRNA-Seq data for human K562 cells that were subjected to heat shock at 42°C for 60 min ^19^. The results showed that heat-shocked cells also had significantly shorter RNAs compared to control cells (Mann–Whitney: p-value < 10^−76^), again independently of differential expression (Fig. 1i, Sup. Fig. 1h, i).

**Fig. 2:**
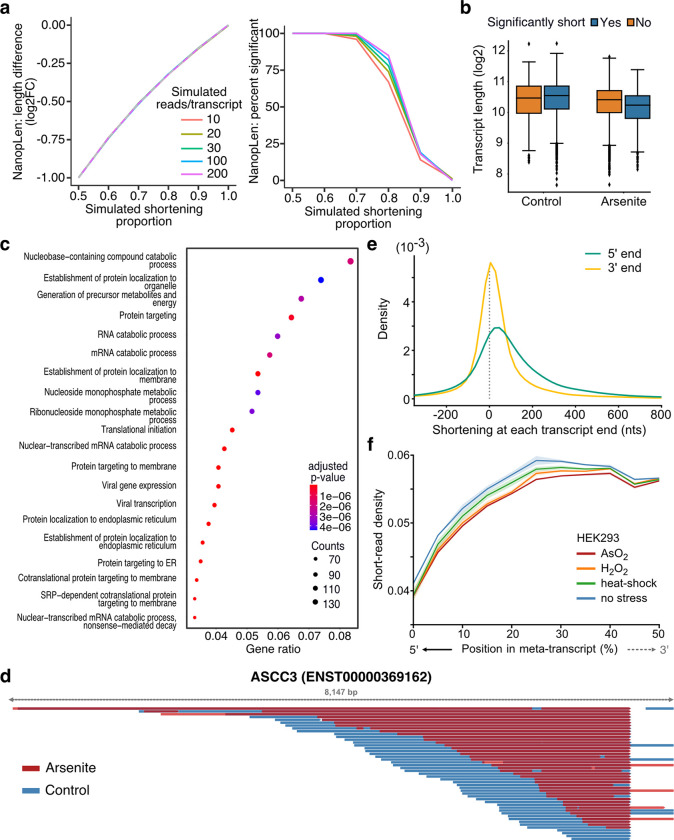
Characterization of OS-induced shortened RNAs identified by NanopLen: **a)** Left: Line plot of average length difference estimate for simulated data of varying read depths. True value is depicted by dashed grey line. Right: Percentage of simulated genes that are detected as significantly different in length. The null simulation of no shortening corresponds to simulated shortening proportion 1.0. **b)** Box plot of average transcript length for statistically significant and non-significantly shortened transcripts identified through differential length analysis in arsenite-treated and control cells using NanopLen. **c)** Gene Ontology analysis for biological processes of significantly shortened transcripts. **d)** IGV screenshot of ASCC3 aligned reads for arsenite-treated (red) and control cells (blue). Libraries were randomly downsampled to maximum 50 reads per window and the libraries were overlayed. All reads were used, irrespective of adaptor ligation status. **e)** Density plot of transcript shortening at the 5’ and 3’ end. Positive values on X-axis indicate that the 5’ or 3’ of transcripts in arsenite-treated cells are correspondingly downstream or upstream of their control counterparts. **f)** Short-read RNA-Seq density at the 5’ half of transcripts for control, AsO_2_, H_2_O_2_ and heat shock -treated cells. Shade indicates standard error of the mean for replicates.

To identify transcripts with systemic RNA length changes, we developed NanopLen, a statistical tool that uses linear mixed models (LMMs) to report statistical significance. We modeled the library as a random effect to adjust for variation across replicates (see methods). To test the model, we simulated sequencing data over varying RNA shortening proportions and expression counts. The simulated data showed that the model accurately predicted the true length difference for each tested shortening proportion and had a well-controlled false positive rate (Fig. 2a). As expected, NanopLen reported lower significance for smaller length differences while higher expression counts resulted in higher statistical power. Applying NanopLen on the data for control and arsenite-treated cells identified 2730 significantly shortened gene transcripts (p-value < 0.05) (Fig. 2b, Sup. Table 4). Gene Ontology (GO) analysis of significantly shortened transcripts identified stress-associated processes relevant to RNA catabolism, translation initiation, and protein localization and targeting to the endoplasmic reticulum (Fig. 2c).

Consistent with the requirement for the presence of poly(A) tail to sequence RNAs through the nanopore, careful inspection of significantly and non-significantly shortened transcripts showed that the 3’ ends of shortened transcripts were almost identical in OS and control whereas the corresponding 5’ ends were more heterogenous (Fig. 2d, Sup. Fig. 2a). In contrast, both ends were identical for non-significantly shortened transcripts (Sup. Fig. 2b). Global quantification at the transcriptome-wide level confirmed this observation showing that 5’ were significantly more shortened and heterogeneous compared to 3’ ends (Mann Whitney p-value < 10^−256^, Fig. 2e). To provide more evidence for this finding we reasoned that RNA shortening at the 5’ end, should also be reflected in short-read RNA-Seq as reduction in read coverage at the 5’ end of RNAs. We thus re-analyzed recently published data from HEK293 cells treated with a battery of stressors i.e. 42 °C heat shock for 1 h; 0.6 mM H_2_O_2_ for 2 h and 300 μM NaAsO_2_ for 2 h followed by short-read RNA-Seq ^29^. Our analysis showed that, compared to control, all stress conditions resulted in significantly reduced read coverage at the 5’ end in agreement with our findings (Fig. 2f, Sup. Fig. 2e). Conclusively our results provide strong evidence for stress-induced decay occurring at the 5’ end of RNA molecules.

### Stress-induced RNA decay is XRN1-mediated but independent of de-adenylation

**Fig. 3:**
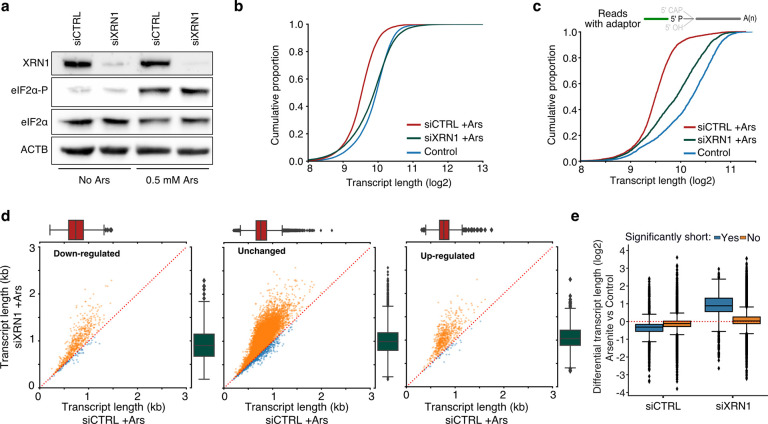
The effect of XRN1 knockdown on RNA shortening. **a)** Immunoblot for XRN1, eIF2α-P and eIF2α for cells transfected with a mock (siCTRL) or *XRN1-*targeting siRNA. ACTB is used as control. **b)** Cumulative distribution plot of transcript length for XRN1 knockdown (siXRN1) and control (siCTRL) in arsenite-treated cells. **c)** Same as (b) but only reads with ligated 5’ end adaptor are used. **d**) Scatter plots of average transcript length for arsenite-treated cells transfected with mock and *XRN1-*targeting siRNA stratified by their differential expression change. Down-regulated: (-Inf, −0.5), Unchanged: (−0.5, 0.5), Up-regulated (0.5, Inf) fold-change. Only transcripts with at least 5 aligned reads are shown. Red dotted indicates the y=x line. Color indicates transcripts below (blue) and above (orange) the diagonal. **e)** Box plots of differential transcript length in arsenite-treated versus control cells for significantly and non-significantly shortened transcripts in XRN1 knockdown and control.

XRN1 is the primary 5’ to 3’ exonuclease in cells, and thus seemed a likely candidate for mediating transcript shortening upon stress. We hypothesized that in the absence of XRN1, RNAs would be stabilized, and their observed length would be mostly restored. We therefore silenced XRN1 with siRNAs (siXRN1) that resulted in substantial reduction of protein level compared to non-targeted control (siCTRL) (Fig. 3a, Sup. Fig. 3a). We subsequently performed TERA-Seq for siCTRL- and siXRN1-transfected cells in the presence or absence of OS. Our results showed that transfection with siXRN1 resulted in significantly longer transcripts than mock transfection largely rescuing RNA length (Fig. 3b). We further verified this result by only examining reads containing a 5’ adaptor, which represent the most confident pool of shortened RNAs (Fig. 3c). Quantifying this length difference at the individual transcript level using all reads showed that indeed most expressed transcripts were generally longer after XRN1 downregulation and this increase was independent of differential expression (Fig. 3d, Sup. Fig. 3b). While silencing of *XRN1* might be expected to have a general effect on RNA length at the 5’ end, we saw that the effects of reduced XRN1 were most prominently observed in the RNAs we identified as shortened during oxidative stress (Fig 3e), strongly implicating both XRN1 and 5’ exonucleolytic decay as the cause of transcript shortening during oxidative stress.

**Fig. 4:**
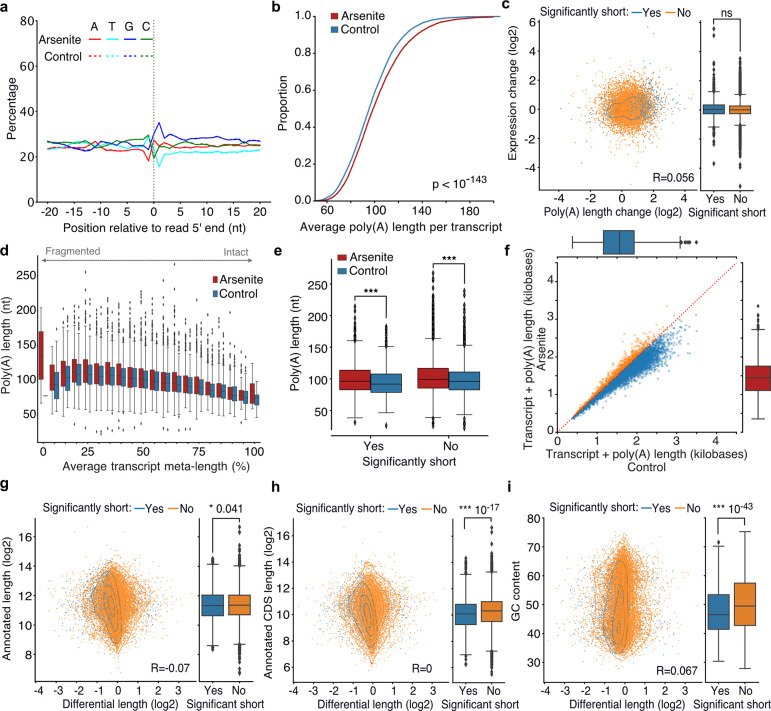
Association of poly(A) tail length and cis-regulatory elements with RNA shortening. **a)** Nucleotide composition around the 5′ end of reads in arsenite-treated and control cells. All reads were used, irrespective of adaptor ligation status. **b)** Cumulative distribution of the average poly(A) tail length per transcript for arsenite-treated and control cells. **c)** Scatter plot of transcript expression against poly(A) tail length differential change in arsenite-treated and control cells. **d)** Box plots of average transcript poly(A) tail length against average transcript-meta length for arsenite-treated and control cells. **e)** Box plots of average transcript poly(A) tail length for significantly and non-significantly shortened transcripts in arsenite-treated and control cells. **f)** Scatter plot of combined average transcript and poly(A) tail length for arsenite-treated and control cells. Only transcripts with at least 5 aligned reads were used. Red dotted indicates the y=x line. Color indicates transcripts below (blue) and above (orange) the diagonal. **g-i)** Scatter plot of annotated transcript length (g), annotated CDS length (h) and GC content (i) against transcript differential length in arsenite-treated and control cells. The box plots on the right side summarize the y-axis variable for significant and non-significantly shortened transcripts. The Pearson’s correlation coefficient and the Mann-Whitney-U test p-value are shown.

The yeast Xrn1 has previously been associated with the decay of oxidized RNAs via the NGD pathway following ribosome stalling at nucleotide adducts, particularly 8-oxoguanosine (8-OxoG), and endonucleolytic cleavage ^22^. We reasoned that if 8-OxoG induced endonucleolytic cleavage were the major contributor of the transcript shortening we observed, then an increase in guanine prevalence should be expected at the vicinity of 5’ ends of sequenced RNAs upon stress. However, our data did not support this explanation, as no G-nucleotide enrichment difference between arsenite and control was observed (Fig. 4a). This finding, combined with the presence of shortening under heat shock (Fig. 1i), indicates that the stress-induced RNA decay described here is unlikely to be mediated via this mechanism.

Under the traditional decay model, de-adenylation is considered the first and rate-limiting step prior to decapping and subsequent exonucleolytic action from the 5’-end via XRN1 ^23^. We therefore wished to assess the possible role of the poly(A) tail in stress-induced RNA decay. We used nanopolish to quantify the poly(A) tail length of the RNA molecules in the TERA-Seq libraries ^24^. Contrary to the traditional model, our data showed a modest but significant increase of poly(A) tail length (p-value < 10^−143^) in arsenite-treated compared to control cells (Fig. 4b). However, no association with gene expression levels was observed (Fig. 4c, Sup. Fig. 4a). To further explore the role of the poly(A) tail, we hypothesized that if de-adenylation was required for RNA shortening then a correlation should be observed between poly(A) tail length and RNA decay levels. Interestingly, we found that the length of poly(A) tails increases in arsenite compared to control regardless of the transcript meta-length (Fig. 4d, e, Sup. Fig. 4b–e). This finding is in agreement with previous studies that have also reported increase of poly(A) tail length in response to endoplasmic reticulum, arsenite, heat, and nutrient starvation stresses ^24^. Finally, we tested a composite metric for transcript length by adding the poly(A) tail length to the corresponding aligned read length. Our data again showed that composite transcript length was reduced in arsenite-treated compared to control cells (Fig. 4f). In conclusion, our results show that stress-induced RNA decay is mediated by XRN1 but is independent of prior de-adenylation.

### Restoring ribosome density inhibits stress-induced RNA decay

To identify putative gene features that could be driving stress-induced RNA decay, we tested whether nucleotide content or the lengths of the coding sequence (CDS), untranslated regions (UTRs), and the total annotated length were predictive of the transcripts subject to stress-induced 5’ shortening. Our results showed no association between the expected length of these features and differential decay, except for the CDS and GC content, that were significantly reduced for significantly shortened transcripts (Fig. 4g–i, Sup. Fig. 4f, g). Since the CDS is the primary region of ribosome occupancy we reasoned that upon stress-induced translation inhibition, shorter CDSs might be leading to faster ribosome run-off that might be contributing to 5’ shortening as the RNAs would be leaving the translational pool. To test this hypothesis, we used publicly available ribosome profiling data ^25^ from unstressed cells. Our analysis showed no association between ribosome density per RNA and shortening (Sup. Fig. 5a, b) indicating that the pre-stress ribosome levels on RNAs are not linked to RNA decay under stress.

**Fig. 5.**
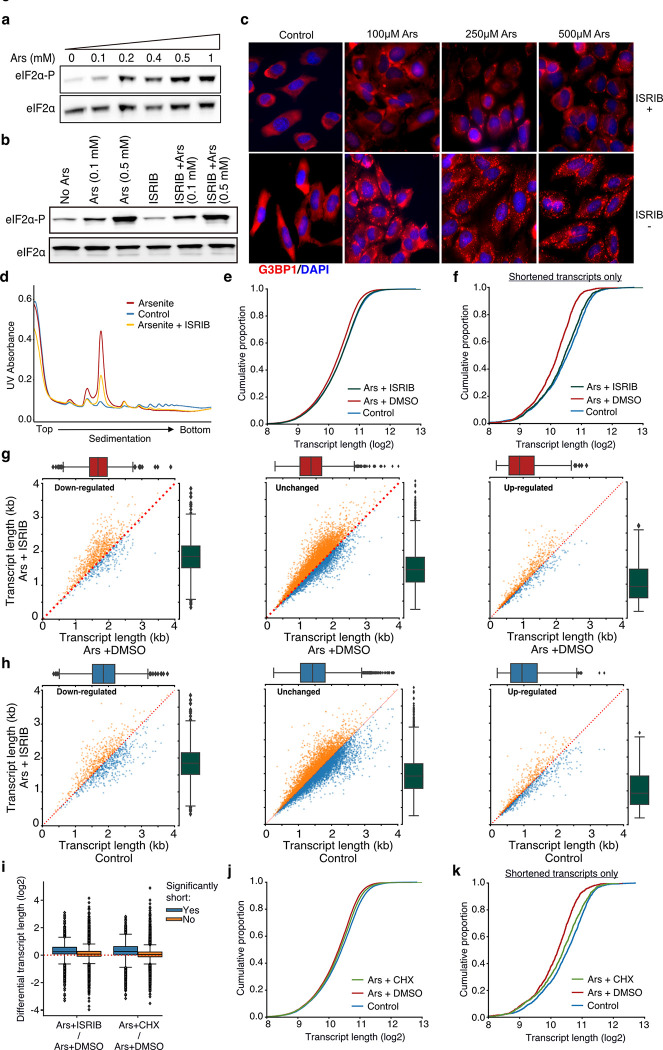
Translation and RNA shortening. **a, b**) Immunoblot for eIF2α and eIF2α-P upon increasing concentration of arsenite (a) and cell treatment with 200 nM of ISRIB at different arsenite concentrations (b). **c)** SGs visualized by immunofluorescence of HeLa cells treated with indicated concentration of arsenite in the presence or absence of 200 nM ISRIB. A secondary goat anti-rabbit IgG H&L (Alexa Fluor 594) against G3BP1 (SG marker) and DAPI were used for visualization. **d)** Ribosome sedimentation curve following cell treatment with ISRIB (200 nM) for arsenite-treated and control cells. **e)** Cumulative density plot of transcript length for arsenite-treated cells in the presence or absence of ISRIB. **f)** Same as (e) for significantly shortened transcripts only. **g)** Scatter plots of average transcript length for arsenite-treated cells with and without ISRIB stratified by their differential expression change. Down-regulated: (-Inf, −0.5), Unchanged: (−0.5, 0.5), Up-regulated (0.5, Inf) fold-change. Only transcripts with at least 5 aligned reads are used. Red dotted indicates the y=x line. Color indicates transcripts below (blue) and above (orange) the diagonal. **h)** Same as (g) for arsenite and ISRIB treated cells against control. **i)** Box plots of differential transcript length for comparisons indicated on the x-axis. **i-k)** Same as (e) and (f) for CHX instead of ISRIB.

Rather, another possibility is that stress-induced RNA decay is associated with the rate at which ribosomes run-off from RNAs. If this was true, then alteration of the ribosome dynamics under stress should have a direct effect on observed RNA decay. Thus, we treated cells with the ISR Inhibitor (ISRIB) which has been shown to bypass the effects of eIF2α phosphorylation and to facilitate translation initiation under stress ^30,31^. Consistent with previous findings, ISRIB had no noticeable effect on the dose-dependent phosphorylation of eIF2α but inhibited the SG formation under stress, as expected (Fig. 5a–c). Polysome fractionation following ISRIB addition showed a clear reduction of the monosome fraction, indicating partial recovery of translation initiation, as previously described ^25^ (Fig. 5d).

We then performed TERA-Seq on ISRIB-treated and control cells in the presence or absence of arsenite to interrogate the effect of ribosome occupancy on RNA decay. Our results showed that compared to cells treated with arsenite only, ISRIB treatment resulted in a significant shift of RNA length towards longer molecules (p-value < 10^−156^) essentially restoring transcript length to the level of the control (Fig 5e). As expected, the previously identified significantly shortened transcripts showed the greatest recovery of their length compared to non-significant ones (Fig 5f). To test whether this could be simply attributed to a possible length imbalance between up- and down-regulated genes we again plotted the average transcript length stratified by the differential expression status. Our data show that the observed shift in length is independent of differential expression status (Fig. 5g, h). As an alternative to ISRIB which modulates translation initiation, we also treated cells with cycloheximide (CHX) to interrogate the dynamics of ribosome elongation and to prevent ribosome run-off. CHX is expected to block elongation and thus “freeze” ribosomes on RNAs. We confirmed that treatment with CHX also reduced the monosome fraction, although to a lower degree than ISRIB (Sup. Fig. 5c). Interestingly, CHX treatment also inhibited RNA decay, particularly for the most significantly shortened RNAs largely rescuing their length (Fig. 5i–k). We again did not observe any association between the shift in length and differential expression (Sup. Fig. 5d, e). Combined our results indicate ribosome occupancy as protective against stress-induced decay while ribosome run-off as a possible contributor to decay.

### Decaying RNAs persist in cells and are enriched in stress granules

**Fig. 6:**
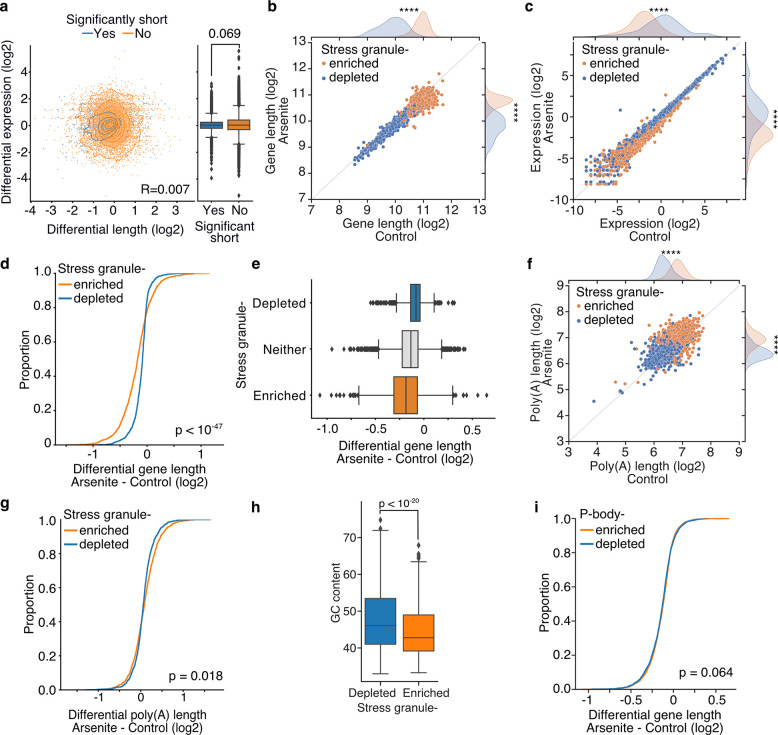
Association of RNA shortening with SG and P-body localization. **a)** Scatterplot of differential length against differential expression in arsenite-treated and control cells stratified for significantly and non-significantly shortened transcripts. **b-c)** Scatter plot of average gene length (b) and upper 90th quantile normalized gene expression (c) for arsenite-treated and control cells stratified by gene SG localization. **d-e)** Cumulative distribution and box plots of average gene length difference in arsenite-treated and control cells stratified by gene SG localization. **f)** Same as (b) for average poly(A) length per gene. **g)** Cumulative distribution of poly(A) tail length for arsenite-treated and control cells stratified by gene SG localization. **h)** Boxplot of GC content percentage for SG enriched and depleted gene transcripts. **i)** Cumulative distribution plot of average gene length difference in arsenite-treated and control cells stratified by gene P-bodies localization. ****, ** indicates p-value < 10^−172^ and < 0.01 respectively.

Our previous analysis has repeatedly showed lack of association between the observed stress-induced decay and transcript abundance. This finding has been unexpected as 5’ end shortening would have been anticipated to result in faster clearance of RNA molecules and therefore decrease in RNA levels. To directly test this, we plotted differential abundance against differential shortening levels and stratified transcripts according to their shortening significance. Surprisingly, we again did not find any correlation between RNA shortening and differential abundance nor any difference for significantly and non-significantly shortened transcripts (Fig. 6a) (Mann–Whitney U test p-value: 0.069). We considered whether a reciprocal increase in transcription rate could be cancelling out changes to expression levels. However, this hypothesis is inconsistent with our data as newly synthesized molecules would have been expected to push the transcript length distribution towards larger lengths; thus, the opposite of what our data show (Fig. 1c–e). Collectively our results indicate that despite the initiation of decay, the decaying RNA fragments persist in cells and raise the possibility of a mechanism that preserves them.

During OS, ribosome run-off and the exit of RNAs from the translation pool is accompanied by the formation of SGs ^17^. We therefore tested whether the decaying transcripts are associated with SGs. We used publicly available data representing genes enriched in the SG transcriptome (Khong et al., 2017) and compared against our dRNA-Seq data. As previously described (Khong et al., 2017), we found that SG-enriched genes were generally significantly longer, particularly in their coding and 3’UTR sequences, (Fig. 6b, Sup. Fig. 6a–d) and less expressed than SG-depleted ones (Fig. 6c). However, differential gene length analysis showed, interestingly, a clear difference in gene shortening dependent on SG enrichment status. Specifically, SG-enriched genes were found to be significantly more shortened upon OS compared to SG-depleted or non-localized RNAs (Fig. 6d, e, Sup. Fig. 6e). We again found no association with poly(A) tail length as, although SG-enriched RNAs had significantly longer poly(A) tails, their lengths did not significantly change upon stress (Fig. 6f, g). Consistent with stress-induced gene shortening being associated with SG enrichment, SG-enriched RNAs harbored lower GC content, similar to significantly shortened transcripts (Fig. 4i, Fig. 6h). To test whether the observed enrichment of shortened genes is specific to the SGs we also tested for association with processing bodies (P-bodies), membraneless organelles enriched in de-capping factors and exoribonucleases, essential for RNA decay under normal, non-stress conditions. We re-analyzed previously published data ^32^, but in contrast to SGs, we found no association between P-body localization and gene lengths (Sup. Fig. 6f). We also did not find an association between P-body localization and RNA shortening (Fig. 6i, Sup. Fig. 6g). Collectively, our results show that shortened RNAs persist in cells and are preferentially localized to the SGs, indicating a possible role of the SGs in the transient storage of these molecules.

## Discussion

Cellular response to stress and the accompanying mechanisms that restore homeostasis are critical for cell survival. Recent works have delineated many of the biochemical steps and factors involved in the stress response but key mechanisms regulating the state of RNA under cellular stress remain unknown. In this work, we used TERA-Seq, a protocol that involves the ligation of unique adaptors at the 5’ end of RNAs ^28^ to address a key limitation in nanopore direct RNA sequencing – the inability to consistently capture the 5’ end of sequenced RNA molecules. By sequencing RNA molecules end-to-end, we acquire a detailed snapshot of the state of RNA upon stress and show that oxidative and heat shock stress trigger decay at the 5′ end of RNAs. Interestingly we find that stress-induced decay also leaves “scars” that can be identified in traditional short-read RNA-Seq reflected as decreased read density at the 5’ end of transcripts that can be important for its interpretation.

Our findings highlight XRN1 as an essential component for stress-induced decay, in the absence of which RNA length is largely rescued. However, contrary to the traditional decay model, evaluation of the most significantly shortened RNAs did not reveal a dependency on de-adenylation. Instead, these shortened transcripts possess shorter CDSs. When exposed to stress, cells enact a widespread halt in translation initiation through eIF2α phosphorylation (Pakos-Zebrucka et al., 2016). This results in a swift detachment of scanning initiation factors from RNAs, eventually leading to ribosome run-off (Bresson et al., 2020). Consequently, our results point towards a model where the occupancy time of ribosomes on RNAs, as determined by the CDS length, could thus play a pivotal role for RNA decay upon stress. Supporting this idea, ISRIB, an ISR inhibitor, re-initiates translation partially and curtails RNA decay. These findings indicate that ribosome run-off is a central factor in the observed RNA shortening under stress.

Interestingly, our end-to-end sequencing data show that the observed RNA shortening is not accompanied by a corresponding change in RNA abundance. This indicates that the RNA state captured in our experiments may correspond to a transient step towards complete RNA clearance. Alternatively, the observed shortened RNAs could be protected from further degradation, presumably by SGs as has previously been suggested ^17^. In line with this idea, our results show that the SG transcriptome is enriched for significantly shortened RNAs. We and others have previously shown that ribonucleoprotein- and stress-granule formation is generally facilitated by stochastic RNA interactions thus, leaning towards an enrichment of longer RNAs ^24,33^. Assuming stress-induced decay relies on SG positioning, the shortened RNAs would be expected to reflect a similar length inclination. However, our data contradict this, implying that SGs aren’t decay centers during stress but likely temporary repositories for partially degraded RNA molecules.

Past research has shown that cells counter stress by broadly halting translation via ISR activation (Pakos-Zebrucka et al., 2016). While the physiological role of stress-induced RNA decay is currently unknown an intriguing hypothesis could be that it evolved as a mechanism to further alleviate the burden on the translation machinery during stress. By breaking down the 5’ end of RNAs and eliminating translation initiation sites, cells may use an orthogonal strategy to comprehensively dial down translation. The mechanistic reason of concentrating these trimmed RNAs remains unclear as the loss of the 5’ end likely deems them unusable for translation after stress recovery. An intriguing hypothesis could be that these RNA fragments might act as sponges for post-transcriptional regulatory factors or simply become reserves for nucleotides post-stress. Future studies to test these hypotheses will be crucial, especially considering the emerging significance of SGs and RNA metabolism in neurodegenerative diseases and aging ^34,35^.

## Materials and Methods

### Cell culture, treatments, and RNA isolation

HeLa cells (ATCC CCL-2) were cultured at 37°C, 5% CO_2_, 90% humidity in Dulbecco’s Modified Eagle Medium (Thermo Fisher Scientific, Cat # 11965–092) supplemented with 10% heat-inactivated fetal bovine serum (GeminiBio #100–106), 2 mM L-Glutamine. The cells were tested for mycoplasma contamination using universal mycoplasma detection kit (ATCC, Cat # 30–1012K). Cells were treated with 500 μM of sodium arsenite (Sigma, Cat # S7400-100G) for 60 minutes and total RNA was isolated using TRIzol reagent (Invitrogen, Cat# 15596-018) following the manufacturer’s recommendation. RNA concentration was quantified using a High Sensitivity (HS) RNA qubit assay (Invitrogen, Cat # Q32852) and Nanodrop ND-1000 (ThermoFisher). RNA integrity was assessed on a 2100 Bioanalyzer (Agilent Technologies) or Qubit^™^ RNA IQ assay (ThermoFisher).

### siRNA transfection

Cells were transfected with control and gene-specific siRNAs at a final concentration of 20 nM using the Neon transfection system 100 μl kit (ThermoFisher, MPK100) according to the manufacturer’s protocol. A commercially available siRNA specifically targeting the human XRN1 was used for XRN1 knockdown, and a non-targeting siRNA was used as control (Sup. Table 2). Approximately 5 × 10^5^ cells were resuspended in 100 μl of siRNA-R buffer mixture, electroporated (1,005 V, 35 ms, 2 pulses) and immediately transferred to a 100 mm dish containing pre-warmed media. Cells were grown for 72 hours before treatment with indicated reagents and final harvesting for RNA and protein assays.

### Direct RNA sequencing

Library preparation was performed using the direct RNA sequencing kit (Oxford Nanopore Technologies, SQK-RNA002) as previously described (Ibrahim et al. 2021) with modifications. A minimum of 75 μg of total RNA was used as starting material to purify poly(A) RNAs using Oligo d(T)25 Magnetic Beads (NEB, Cat # s1419S). 50 pmoles of linker (REL5) containing a 5′-Biotin-PC group and a 3’-OH (Sup. Table. 2) were ligated to the 5′ end of RNAs using T4 RNA ligase (NEB, Cat #M0204S) for 3 hours at 37°C, as previously described ^28^. 500–1000 ng of poly(A) RNA was used for library preparation using SQK-RNA002 sequencing kit (Oxford Nanopore Technologies). The final library was quantified using Qubit 1X dsDNA High Sensitivity (HS) assay kit (ThermoFisher # Q33231) and loaded on FLO-MIN106 or FLO-PRO002 flowcells.

### MTS assay

MTS assay was performed using CellTiter 96 AQueous One Solution Cell Proliferation Assay (MTS) (Promega, Cat # G3582). HeLa cells (5 × 10^3^ cells per well) were seeded in a 96-well plate and incubated in humidified 5% CO_2_ incubator at 37°C for 24 hours. Following arsenite treatment, the cell viability was examined using MTS assay (100 μl of DMEM and 20 μl MTS incubated for indicated time points) for 10 minutes and incubated in a 5% CO_2_ incubator at 37 °C protected from light. Finally, the plate was subjected to shaking for 15 min followed by an optical density measurement (OD) at 590 nm using 1420 Multilabel Counter (Perkin Elmer, VICTOR^3^V). The viable cells in arsenite treated samples were reported as the percentage of viable cells in control (untreated) samples.

### Immunoblotting

Cells were washed with cold PBS and pelleted by centrifugation 5 min at 4 °C 300 *× g*. Whole cell lysates were prepared by adding 1 volume of SDS lysis buffer (60 mM Tris-HCl pH 7.5, 2% SDS, 10% glycerol) supplemented with 1x protease inhibitor cocktail (Roche Diagnostics, Cat # 11836153001) and 1 mM phenylmethylsulfonyl fluoride (PMSF) (Roche, Cat # 10837091001). The lysate was passed 10 times through a 25-gauge needle, heated at 95°C for 20 minutes and cleared by centrifugation at 16,500 *× g* for 10 minutes. Protein concentrations were measured using the Qubit protein assay (Invitrogen, Cat # Q33211). 25 μg of proteins was separated on NuPAGE 4–12% Bis-Tris Gel (Invitrogen, Cat #NP0321BOX) and transferred to PVDF membrane (Millipore, Cat # IPVH00010). After blocking in TBS-T 5% milk for 2 hours, membranes were incubated with primary antibodies overnight at 4°C. Membranes were washed 3 times 5 min in TBS-T and incubated for 2 hours with secondary antibodies. Signals were developed using Chemiluminescence (Azure Biosystems, Cat # AC2204) and acquired on a ChemiDoc MP imaging system (Biorad). For studies of eIF2α phosphorylation (eIF2α-P), the lysis buffer was supplemented with phosphatase inhibitors (Sigma, Cat # Cocktail 2 - P5726 and Cocktail 3-P0044) and 5% BSA was used instead of milk for blocking. Antibodies are listed in Sup. Table 2.

### Immunofluorescence

Cells were cultured at 70–80% confluency in Millicell EZ SLIDE 8-well glass (Milipore, Cat # PEZGS0816) in the presence of 50, 100, 250, 500 μM of arsenite +/− 200 nM of ISRIB (Sigma, Cat # SML0843). After the indicated time of treatment, cells were washed 3 times with warm PBS, fixed for 10 min at room temperature (RT) in PBS / 3.7% paraformaldehyde and washed with PBS for 5 min with shaking. After permeabilization in PBS 0.5% Triton X-100 for 10 min at RT, cells were blocked in PBS 0.1% Tween-20 (PBS-T) supplemented with 1% BSA for 1 hour at 37°C with gentle shaking. Primary antibody diluted in 1% BSA were added to the cells and incubated at 4C overnight. Cells were washed in PBS-T three times 5 min with shaking before staining with secondary antibody for 1 hour at RT. Cells were washed four times for 5 min with PBS-T with shaking and nuclei were stained with 4,6-diamidino-2-phenylindole (DAPI) (1:1000 in PBS-T) for 5 min at RT. Slides were mounted with ProLong Glass Antifade Mountant (Invitrogen, Cat # P36982) and images were acquired using a DeltaVision Microscope System (Applied Precision).

### Polysome profiling

The polysome profiling was performed as described previously ^36^ with modifications. Briefly, HeLa cells (80–85% confluency) cultured in 100 mm dishes were treated with 500 μM sodium arsenite, 200 nM ISRIB or 25 μg/ml CHX as indicated. DMSO was used as a control. Immediately before harvesting, cells were treated with 100 μg/ml CHX for 10 minutes. After one wash with ice-cold PBS containing 100 μg/ml CHX, 500 μL of polysome extraction buffer (20 mM Tris-HCl pH 7.5, 50 mM NaCl, 50 mM KCl, 5 mM MgCl_2_, 1mM DTT, 1 X HALT Protease, 1.0% Triton x-100, and 100 μg/ml CHX) was added directly to the plate and lysates were harvested by scraping. The lysate was cleared by centrifugation at 14,000 *× g* for 10 min. The supernatant was loaded onto the 10–50% sucrose gradient followed by high-speed centrifugation (260,800 *× g* for 90 minutes at 4°C). Using a density gradient fractionation system monitored by UV absorbance detector (A254), 12 fractions were collected.

### Nanopore sequencing data processing

Nanopore sequencing data were basecalled using Guppy (v3.4.5). The 5′ adaptors were identified and removed using cutadapt (v2.8). Reads were first aligned against ribosomal sequences obtained from SILVA ^37^. Non-ribosomal reads were subsequently mapped against the human genome hg38 using minimap2 (version 2.17) ^38^ and parameters -a -x splice -k 12 -u b -p 1 --secondary=yes. They were also aligned against the human transcriptome using -a -x map-ont -k 12 -u f -p 1 --secondary=yes.

### Poly(A) tail length estimation

The poly(A) tail lengths were extracted from sequenced reads using the nanopolish polya package (Workman et al., 2019). Only the poly(A) tail lengths which passed the software quality control scores and were tagged as “PASS” were used in our analysis.

### Differential gene expression and Gene Ontology

Transcript counts were quantified from the transcriptome alignments as the total number of reads aligning on each transcript. Differential expression analysis was performed using DESeq2 ^39^. EnhancedVolcano (https://github.com/kevinblighe/EnhancedVolcano) was used for visualization. GO analysis was performed for significantly changed genes (p-value< 0.05) using “clusterProfiler” ^40^.

### Ribosome profiling data

Ribosome profiling sequencing data were downloaded from GEO GSM2100595 (Park et al., 2016). Adaptors were removed using cutadapt (version 2.8) and reads were aligned to the human genome using STAR (2.5.3a). Further processing to calculate counts per transcript was performed with in-house scripts using pysam (v0.15.4) and counts were converted to reads per kilobase of transcript per million (RPKM).

### Meta-length calculation

For calculating the bin (meta) lengths, we divided the transcripts into 20 equal bins [0, 19]. Reads were assigned to each of these bins based on the corresponding location of their ends. The binned- (meta-) length is calculated as the difference of the binned 3’ end over the binned 5’ end.

### Transcription Start Site identification

Transcription start site identification was performed as previously described ^28^. Briefly, all reads from all replicates were combined and the distribution of read 5’ ends within the 5’ UTR was quantified. The TSS was selected as the position with the highest read density withing the 5’ UTR with minimum 5 supporting reads.

### NanopLen

NanopLen reads a file of read lengths with library identifiers and gene or transcript identifiers, and a metadata file describing the experimental design. The latter includes the library identifiers and the corresponding condition for each library. The software supports three models: t-test, Wilcoxon, and linear mixed model (LMM). In the t-test and LMM options, the user can also supply a customized model to use extra variables in the metadata. In this work we have used the LMM to adjust for putative batch effects across libraries.

Given a vector Y of read lengths associated with a gene/transcript, the t-test is functionally equivalent to a linear regression model. The model also supports optional read-specific extra covariates but are not used in this work:

$$Y=\beta_{0}+\beta_{cond}cond+\epsilon +\left(\text{extra}\;\text{covariates}\right).$$

Similarly, the LMM models Y using the condition as fixed effect and the library identifier as a random effect.

$$Y=\beta_{0}+\beta_{cond}cond+lib+\epsilon +\left(\text{extra}\;\text{covariates}\right),$$

where lib is the random effect of each library. By adding the random effect, the analysis is more robust against potential false positives from library variation and prevents deeper libraries from dominating the effect size. A Wald test is used to test for significance of $\beta_{cond}$. The Wilcoxon test option is restricted to only testing the condition variable and cannot adjust for additional variables. Each gene/transcript is assigned a statistic corresponding to the test used and a p-value. p-values are adjusted for multiple testing using the Bonferroni-Hochberg method.

To test NanopLen models, transcript length data with known shortening rates were simulated. Each simulation was parameterized with a “true” transcript length, a read count as a proxy for transcript expression, and a shortening proportion. The database of known human genic lengths from Ensembl (release 91) was used and the median was selected as “true” length. Since the simulated variance is in proportion of the true length, using any length will result in similar results so we do not simulate additional true lengths. Expression counts were simulated, ranging from 10 to 200 reads per transcripts to capture the dynamic expression range particularly towards small read depth. Varying shortening percentages were simulated from 50, 60, .., 100% of the true length (equivalent of −1, −0.74, .., 0 log2 fold change) with the 100% corresponding to the null simulation of no-change.

Given the parameters, the simulated lengths were generated based on the mixed model in NanopLen, with the number of sampled lengths being the selected expression counts. The library random effect was simulated as if imitating a fluctuation with standard deviation of 10% of the true length, and the global error having a standard deviation of 20% of the expected length mean. As with the real experiment, six libraries of three control and three condition were simulated, with 1000 genes per scenario. Using the LMM option with the logscale option selected, the resulting log2FCs and p-values were calculated and aggregated in Sup. Fig. 2a.

NanopLen is available in https://github.com/maragkakislab/nanoplen

### IGV read density plots

Transcripts that were identified as significantly shortened at a false discovery rate of 0.05 by NanopLen, had at least 50 supporting reads in both conditions and had a difference of 200 nts in length were selected. From the list of 32 transcripts, 5 were randomly selected (*ASCC3*, *FASTKD2*, *MTRR, HNRNPM* and *DSG2*) for visualization. As control, 3 transcripts with non-significant, lower than 10 nt difference (*AGPAT2*, *EPHX1*, *TBCA*) were selected. Libraries were randomly downsampled to maximum 50 reads per window and the libraries were overlayed in Affinity Designer. All reads were used, irrespective of adaptor ligation status.

## Supplementary Material

Supplement 1

Supplement 2

Supplement 3

Supplement 4

Supplement 5

## Data Availability

Sequencing data have been deposited in the Gene Expression Omnibus (GEO); accession: GSE204785.
