# Supplementary material for: Full-length direct RNA sequencing uncovers stress-granule dependent RNA decay upon cellular stress
Source: bioRxiv. 2024 Jan 26:2023.08.31.555629. Originally published 2023 Sep 1. Preprint. [Version 2] doi: 10.1101/2023.08.31.555629 (PMC10491209; doi:10.1101/2023.08.31.555629)
Supplement: Supplementary file 1 [file NIHPP2023.08.31.555629v2-supplement-5.pdf]

bioRxiv preprint doi: https://doi.org/10.1101/2023.08.31.555629; this version posted January 26, 2024. The copyright holder for this preprint (which was not certified by peer review) is the author/funder. This article is a US Government work. It is not subject to copyright under 17 USC 105 and is also made available for use under a CC0 license.

Supplementary Figures

Supplementary Figure 1

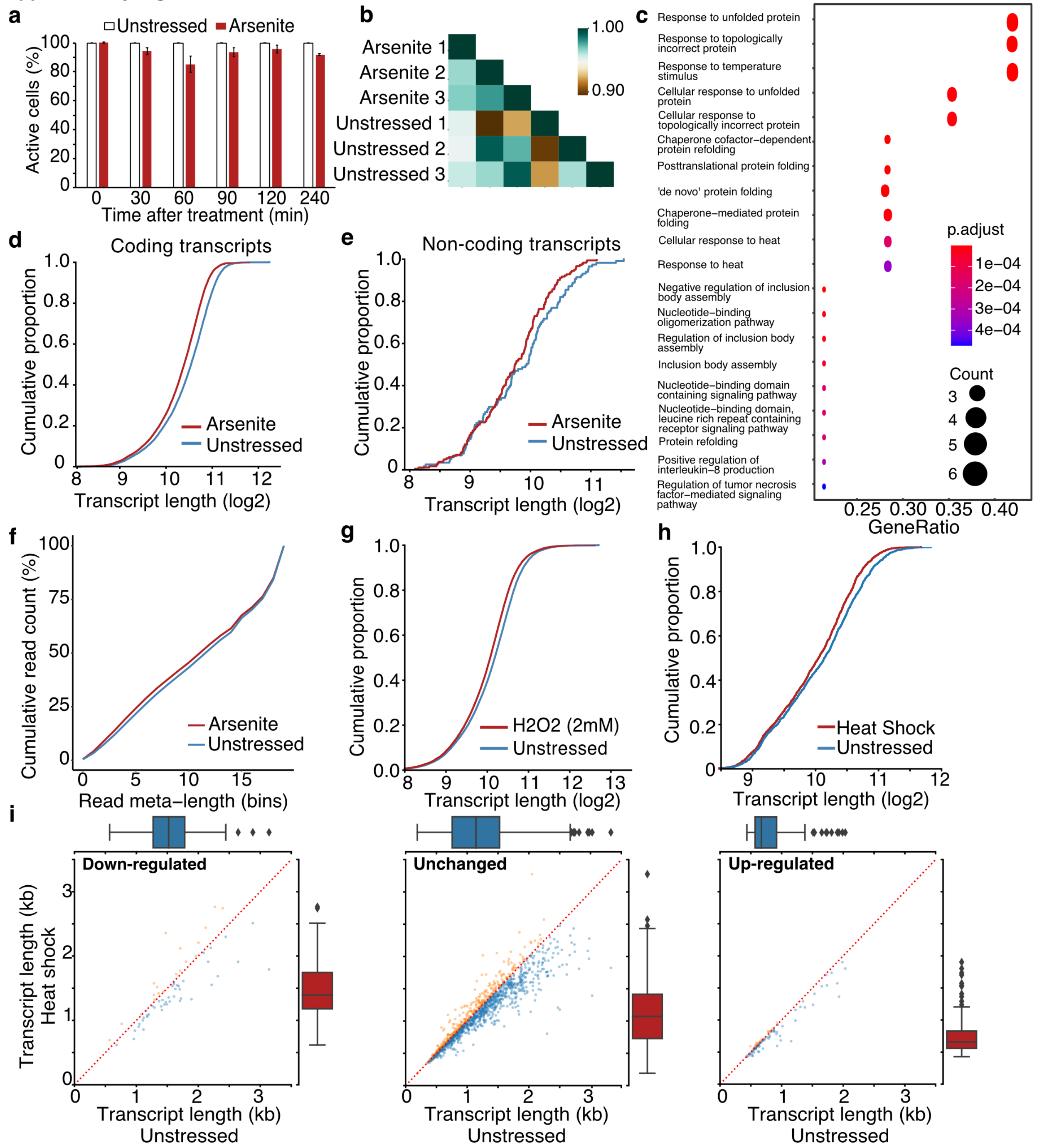

Sup. Fig 1: RNA shortening upon cellular stress. a) MTS cell proliferation assay showing the percentage of active cells (y-axis) upon arsenite treatment compared to unstressed at different time points (x-axis). Error bars represent std for biological replicates (n = 2). **b)** Correlogram of transcript expression for unstressed and arsenite-treated cells. **c)** Gene ontology for biological processes for differentially expressed genes in arsenite-treated versus unstressed cells. **d-e)** Cumulative distribution plot of average transcript length for coding (d) and non-coding (e)

bioRxiv preprint doi: https://doi.org/10.1101/2023.08.31.555629; this version posted January 26, 2024. The copyright holder for this preprint (which was not certified by peer review) is the author/funder. This article is a US Government work. It is not subject to copyright under 17 USC 105 and is also made available for use under a CC0 license.

transcripts. Transcripts with less than 5 reads have been removed. **f)** Cumulative distribution of read meta-length for arsenite-treated and unstressed cells. **g)** Cumulative distribution plot of average transcript length for H2O2-treated cells and unstressed. **h)** Cumulative distribution plot of average transcript length for heat shock cells and unstressed. **i)** Scatter plots of average transcript length for heat shock against unstressed cells stratified by differential expression change. Down-regulated: (-Inf, -0.5), Unchanged: (-0.5, 0.5), Up-regulated (0.5, Inf) fold-change. Only transcripts with at least 5 aligned reads are shown. Red dotted indicates the y=x line. Color indicates transcripts below (blue) and above (orange) the diagonal.

bioRxiv preprint doi: https://doi.org/10.1101/2023.08.31.555629; this version posted January 26, 2024. The copyright holder for this preprint (which was not certified by peer review) is the author/funder. This article is a US Government work. It is not subject to copyright under 17 USC 105 and is also made available for use under a CC0 license.

Supplementary Figure 2

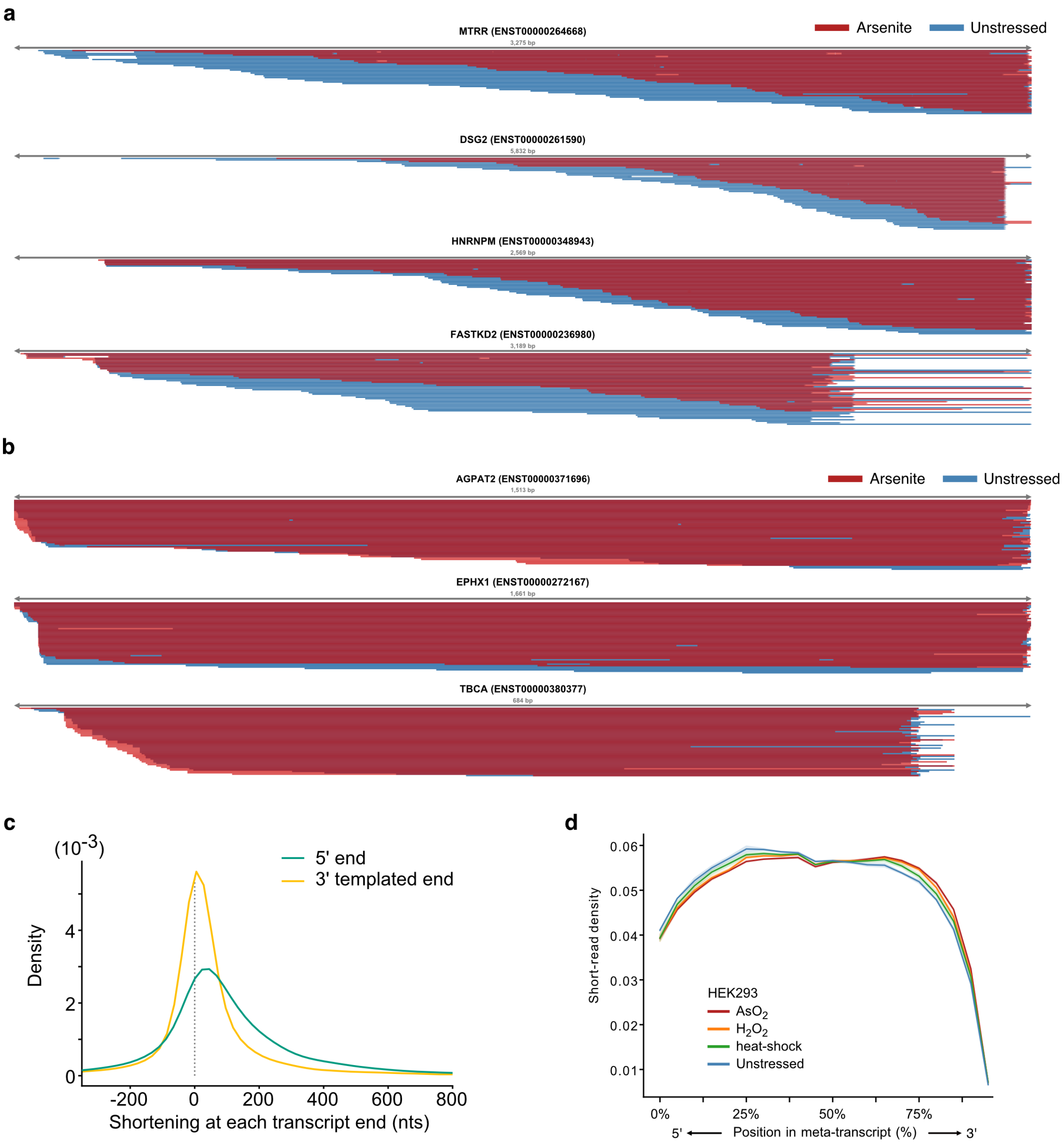

Sup. Fig. 2: Characterization of stress-induced shortened RNAs identified by NanopLen. a) IGV screenshot of aligned reads in arsenite-treated (red) and unstressed cells (blue) for significantly shortened transcripts *FASTKD2*, *HNRNPM*, *DSG2* and *MTRR*. Libraries were randomly downsampled to maximum 50 reads per window and the libraries were overlayed. All reads were used, irrespective of adaptor ligation status. **b)** Same as (a) but for non-significantly shortened transcripts *AGPAT2*, *EPHX1* and *TBCA*. **c)** Density plot of transcript shortening at the 5' and 3' templated end. Positive values on X-axis indicate that the 5' or 3' of transcripts in arsenite-treated cells are correspondingly downstream or upstream of their unstressed

bioRxiv preprint doi: https://doi.org/10.1101/2023.08.31.555629; this version posted January 26, 2024. The copyright holder for this preprint (which was not certified by peer review) is the author/funder. This article is a US Government work. It is not subject to copyright under 17 USC 105 and is also made available for use under a CC0 license.

counterparts. **d)** Meta-plot of short-read RNA-Seq density on all transcripts for unstressed, AsO_2, H_2O_2 and heat shock -treated cells. Shade indicates standard error of the mean for replicates.

bioRxiv preprint doi: https://doi.org/10.1101/2023.08.31.555629; this version posted January 26, 2024. The copyright holder for this preprint (which was not certified by peer review) is the author/funder. This article is a US Government work. It is not subject to copyright under 17 USC 105 and is also made available for use under a CC0 license.

Supplementary Figure 3

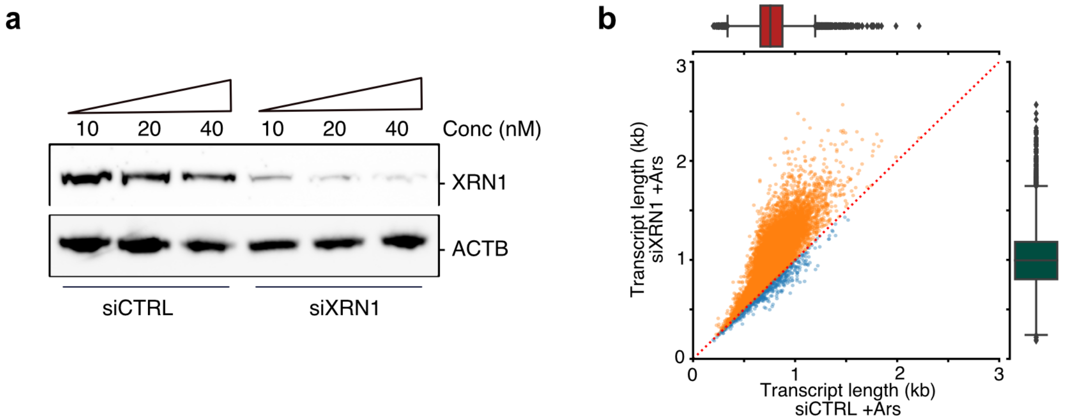

Sup. Fig. 3. The effect of XRN1 knockdown on RNA shortening. a) Immunoblot for XRN1 for cells transfected with mock and *XRN1*-targeting siRNA at varying concentrations. ACTB is used as control. **b)** Scatter plot of average transcript length for XRN1 knockdown and control in arsenite-treated cells. Only transcripts with at least 5 aligned reads are shown. Red dotted indicates the y=x line. Color indicates transcripts below (blue) and above (orange) the diagonal.

bioRxiv preprint doi: https://doi.org/10.1101/2023.08.31.555629; this version posted January 26, 2024. The copyright holder for this preprint (which was not certified by peer review) is the author/funder. This article is a US Government work. It is not subject to copyright under 17 USC 105 and is also made available for use under a CC0 license.

Supplementary Figure 4

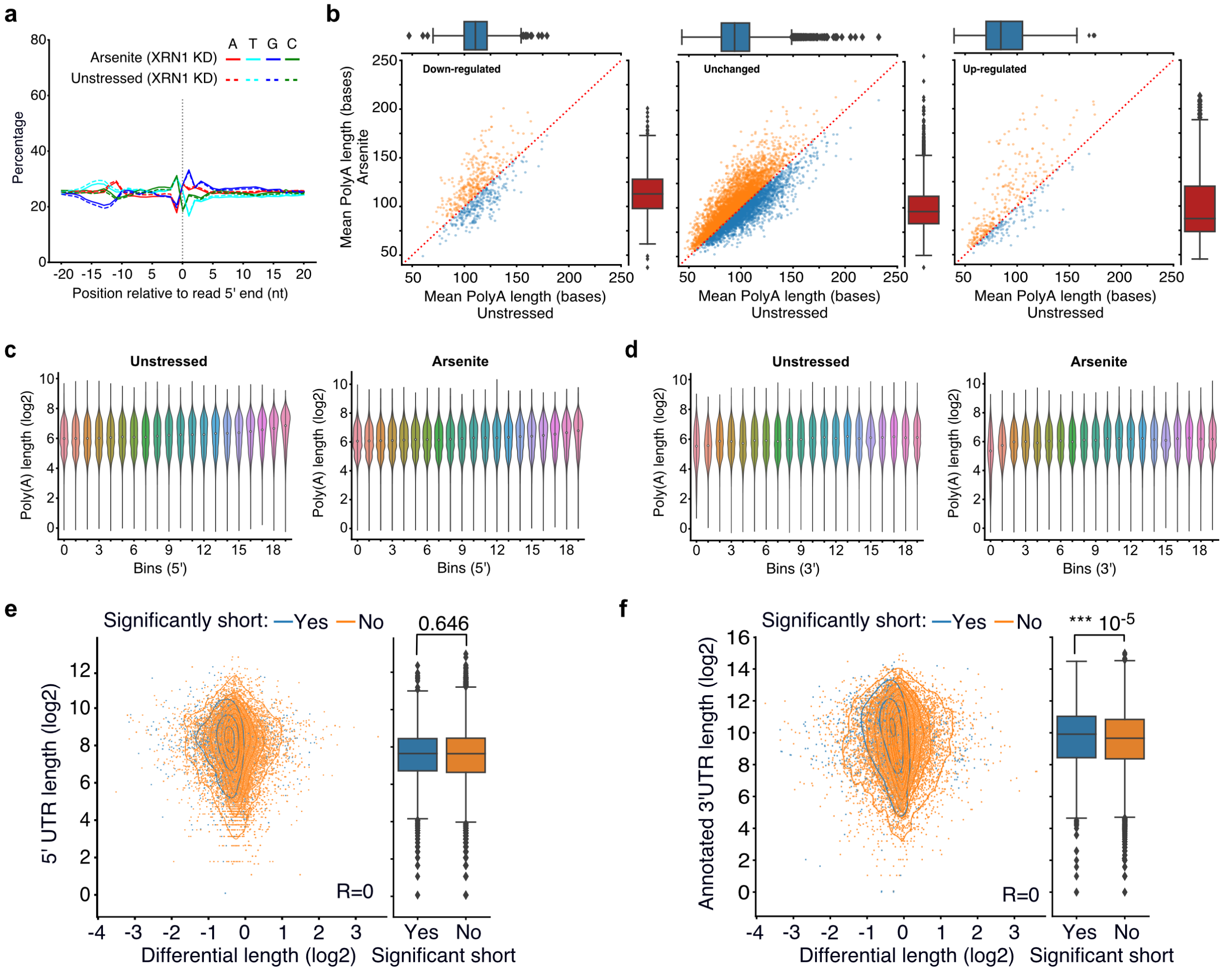

Sup. Fig. 4: Association of poly(A) tail length and cis-regulatory elements with RNA shortening. a) Nucleotide composition around the 5′ end of reads in arsenite-treated and unstressed cells upon XRN1 silencing. All reads were used, irrespective of adaptor ligation status. **b)** Scatter plots of average poly(A) tail length for arsenite-treated and unstressed cells stratified by differential expression. Down-regulated: (-Inf, -0.5), Unchanged: (-0.5, 0.5), Up-regulated (0.5, Inf) fold-change. Only transcripts with at least 5 aligned reads are shown. Red dotted indicates the y=x line. Color indicates transcripts below (blue) and above (orange) the diagonal. **c-d)** Violin plots of average transcript poly(A) length and 5′ or 3′ meta-coordinates for arsenite-treated and unstressed cells. **e-f)** Scatter plot for 5′ (e) and 3'UTR (f) length of annotated transcripts against transcript differential length in arsenite-treated and unstressed cells. The box plots on the right side summarize the y-axis variable for significant and non-significantly shortened transcripts. The Pearson's correlation coefficient and the Mann-Whitney-U test p-value are shown.

bioRxiv preprint doi: https://doi.org/10.1101/2023.08.31.555629; this version posted January 26, 2024. The copyright holder for this preprint (which was not certified by peer review) is the author/funder. This article is a US Government work. It is not subject to copyright under 17 USC 105 and is also made available for use under a CC0 license.

Supplementary Figure 5

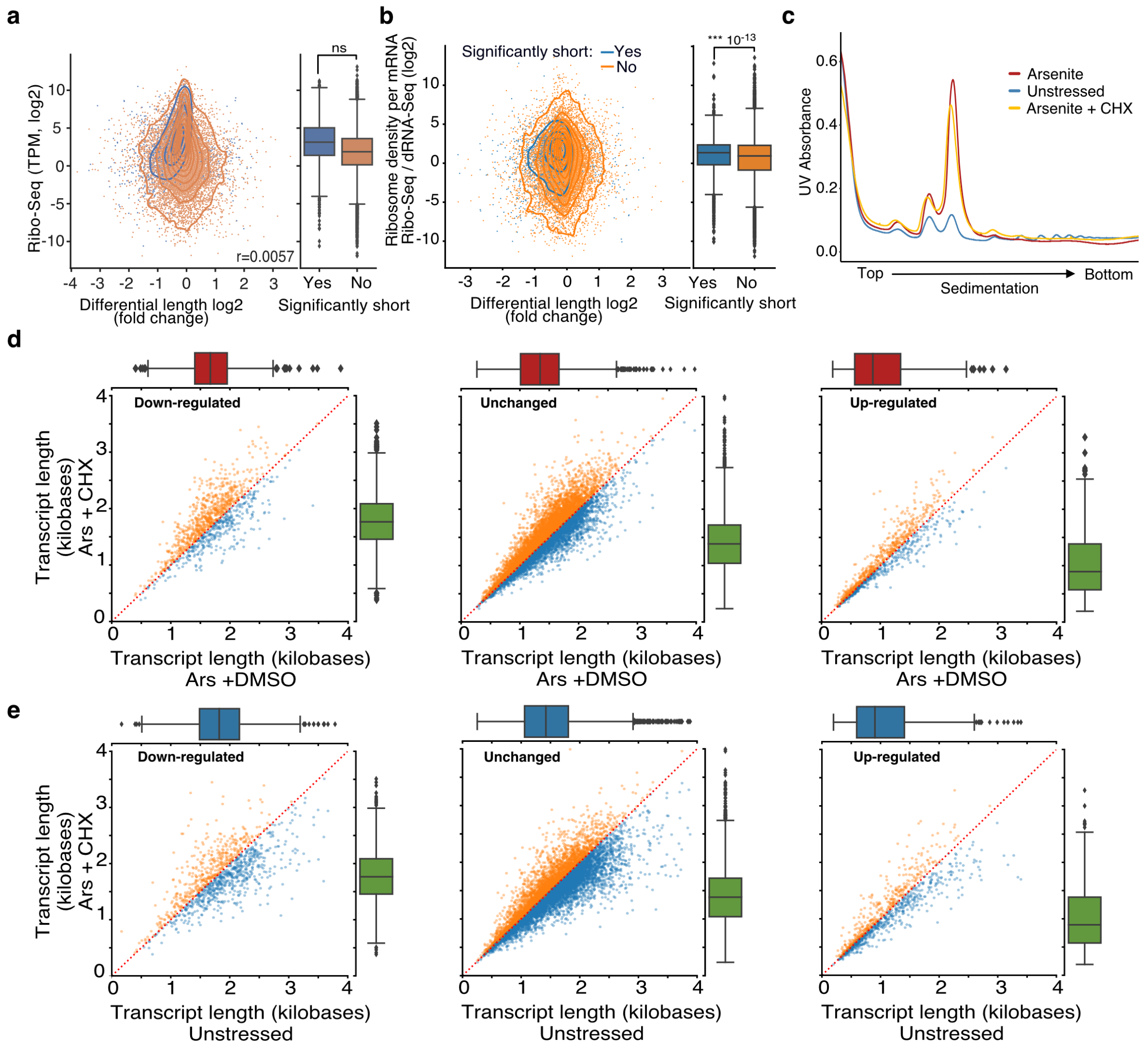

Sup. Fig. 5. Translation and RNA shortening. a-b) Scatter plot of ribosome profiling footprint (a) and translational efficiency (b) levels against differential length in arsenite-treated and unstressed cells. Transcripts are stratified by significance of shortening **c)** Ribosome sedimentation curve following cell treatment with cycloheximide (CHX) (25 µg/ml) for arsenite-treated and unstressed cells. **d, e)** Scatter plots of average transcript length for arsenite plus cycloheximide treated cells against arsenite plus DMSO (d) and unstressed cells (e) stratified by their differential expression change. Down-regulated: (-Inf, -0.5), Unchanged: (-0.5, 0.5), Up-regulated (0.5, Inf) fold-change. Only transcripts with at least 5 aligned reads are shown. Red dotted indicates the y=x line. Color indicates transcripts below (blue) and above (orange) the diagonal.

bioRxiv preprint doi: https://doi.org/10.1101/2023.08.31.555629; this version posted January 26, 2024. The copyright holder for this preprint (which was not certified by peer review) is the author/funder. This article is a US Government work. It is not subject to copyright under 17 USC 105 and is also made available for use under a CC0 license.

Supplementary Figure 6

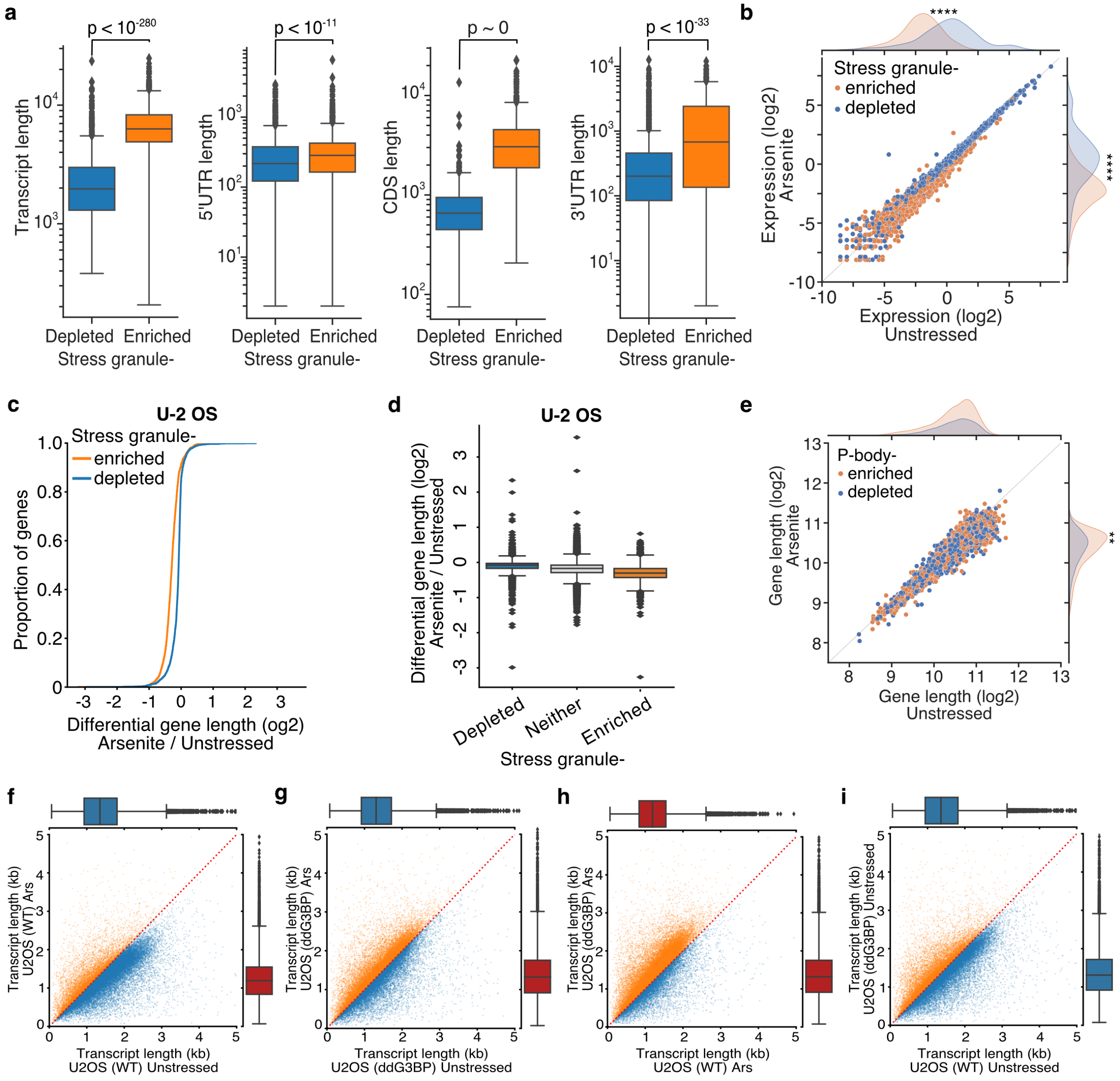

Sup. Fig 6: Inhibition of stress-granule formation in cells devoid of G3BP1/2 rescues RNA decay. a) Box plots of annotated total, 5′ UTR, coding sequence and 3′ UTR length for SG enriched and depleted transcripts. **b)** Scatter plot of upper 90th quantile normalized gene expression for arsenite-treated and unstressed cells stratified by gene SG localization. **** indicates p-value < 10^{-172}. **c)** Cumulative density plot of transcript differential length for SG enriched and depleted gene transcripts in U-2 OS cells. Only transcripts of coding genes are used. **d)** Box plots of differential gene length in arsenite-treated and unstressed cells stratified by SG enrichment in U-2 OS cels. **e)** Scatter plot of average gene length for arsenite-treated and unstressed cells stratified by gene P-bodies localization. ** indicates p-value < 0.01. **f-i)** Scatter plot of average transcript length for arsenite-treated and unstressed U-2 OS and ΔΔG3BP1/2 cells. Only transcripts with at least 5 aligned reads are used. Red dotted indicates the y=x line. Color indicates transcripts below (blue) and above (orange) the diagonal.
