# Supplementary material for: Full-length direct RNA sequencing reveals widespread RNA decay upon cellular stress
Source: bioRxiv. 2023 Sep 1:2023.08.31.555629. Preprint. [Version 1] doi: 10.1101/2023.08.31.555629 (PMC10491209; doi:10.1101/2023.08.31.555629)

bioRxiv preprint doi: https://doi.org/10.1101/2023.08.31.555629; this version posted September 1, 2023. The copyright holder for this preprint (which was not certified by peer review) is the author/funder. This article is a US Government work. It is not subject to copyright under 17 USC 105 and is also made available for use under a CC0 license.

Sup. Fig 1: RNA shortening upon cellular stress. **a)** MTS cell proliferation assay showing the percentage of active cells (y-axis) upon arsenite treatment samples compared to untreated (control) at different time points (x-axis). Error bars represent std for biological replicates (n = 2). **b)** Correlogram of transcript expression for control and arsenite-treated cells. **c)** Gene ontology for biological processes for differentially expressed genes in arsenite-treated versus control cells. **d-e)** Cumulative distribution plot of average transcript length for coding (d) and non-coding (e) transcripts. Transcripts with less than 5 reads have been removed. **f)** Cumulative distribution of read meta-length for arsenite-treated and control cells. **g)** Cumulative distribution plot of average transcript length for H2O2-treated cells and control. **h)** Cumulative distribution plot of average transcript length for heat shock cells and control. **i)** Scatter plots of average transcript length for heat shock against control cells stratified by differential expression change. Down-regulated: (-Inf, -0.5), Unchanged: (-0.5, 0.5), Up-regulated (0.5, Inf) fold-change. Only transcripts with at least 5 aligned reads are shown. Red dotted indicates the y=x line. Color indicates transcripts below (blue) and above (orange) the diagonal.

Sup. Fig. 2: Characterization of OS-induced shortened RNAs identified by NanopLen. a) IGV screenshot of aligned reads in arsenite-treated (red) and control cells (blue) for significantly shortened

bioRxiv preprint doi: https://doi.org/10.1101/2023.08.31.555629; this version posted September 1, 2023. The copyright holder for this preprint (which was not certified by peer review) is the author/funder. This article is a US Government work. It is not subject to copyright under 17 USC 105 and is also made available for use under a CC0 license.

transcripts *FASTKD2*, *HNRNPM*, *DSG2* and *MTRR*. Libraries were randomly downsampled to maximum 50 reads per window and the libraries were overlayed. All reads were used, irrespective of adaptor ligation status. **b)** Same as (a) but for non-significantly shortened transcripts *AGPAT2*, *EPHX1* and *TBCA*. **c)** Meta-plot of short-read RNA-Seq density on all transcripts for control, AsO_2, H_2O_2 and heat shock -treated cells. Shade indicates standard error of the mean for replicates.

Sup. Fig. 3. The effect of XRN1 knockdown on RNA shortening. a) Immunoblot for XRN1 for cells transfected with mock and *XRN1*-targeting siRNA at varying concentrations. ACTB is used as control. **b)** Scatter plot of average transcript length for XRN1 knockdown and control in arsenite-treated cells. Only transcripts with at least 5 aligned reads are shown. Red dotted indicates the y=x line. Color indicates transcripts below (blue) and above (orange) the diagonal.

Sup. Fig. 4: Association of poly(A) tail length and cis-regulatory elements with RNA shortening. a) Scatter plots of average poly(A) tail length for arsenite-treated and control cells stratified by differential expression. Down-regulated: (-Inf, -0.5), Unchanged: (-0.5, 0.5), Up-regulated (0.5, Inf) fold-change. Only transcripts with at least 5 aligned reads are shown. Red dotted indicates the y=x line. Color indicates transcripts below (blue) and above (orange) the diagonal. **b-e)** Violin plots of average transcript poly(A) length and 5′ or 3′ meta-coordinates for arsenite-treated and control cells. **f-g)** Scatter plot for 5′ (f) and 3'UTR (g) length of annotated transcripts against transcript differential length in arsenite-treated and control cells. The box plots on the right side summarize the y-axis variable for significant and non-significantly shortened transcripts. The Pearson's correlation coefficient and the Mann-Whitney-U test p-value are shown.

Sup. Fig. 5. Translation and RNA shortening. a-b) Scatter plot of ribosome profiling footprint (a) and translational efficiency (b) levels against differential length in arsenite-treated and control cells. Transcripts are stratified by significance of shortening **c)** Ribosome sedimentation curve following cell treatment with CHX (25 μg/ml) for arsenite-treated and control cells. **d, e)** Scatter plots of average transcript length for arsenite plus ISRIB treated cells against arsenite plus DMSO (d) and control cells (e) stratified by their differential expression change. Down-regulated: (-Inf, -0.5), Unchanged: (-0.5, 0.5), Up-regulated (0.5, Inf) fold-change. Only transcripts with at least 5 aligned reads are shown. Red dotted indicates the y=x line. Color indicates transcripts below (blue) and above (orange) the diagonal.

Sup. Fig 6: Association of RNA shortening with SG and P-body localization. a-d) Box plots of annotated total (a), 5′ UTR (b), coding sequence (c) and 3′ UTR length (d) for SG enriched and depleted transcripts. **e)** Cumulative density plot of transcript differential length for SG enriched and depleted gene transcripts. Only transcripts of coding genes are used **f)** Scatter plot of average gene length for arsenite-treated and control cells stratified by gene P-bodies localization. **g)** Box plot of average gene length in arsenite-treated and control cells and P-bodies-enriched and -depleted gene transcripts.

bioRxiv preprint doi: https://doi.org/10.1101/2023.08.31.555629; this version posted September 1, 2023. The copyright holder for this preprint (which was not certified by peer review) is the author/funder. This article is a US Government work. It is not subject to copyright under 17 USC 105 and is also made available for use under a CC0 license.

Supplementary Figure 1

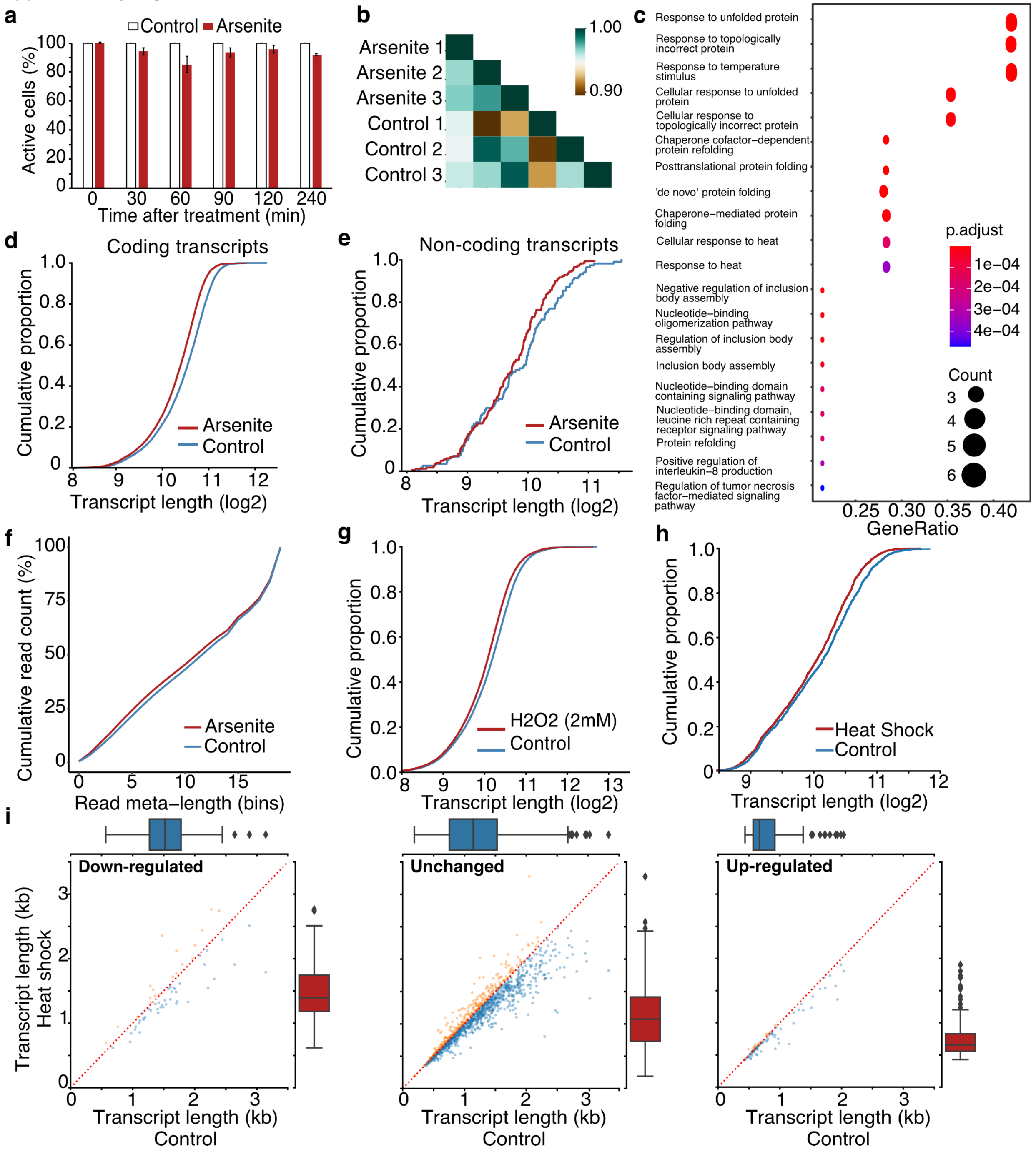

bioRxiv preprint doi: https://doi.org/10.1101/2023.08.31.555629; this version posted September 1, 2023. The copyright holder for this preprint (which was not certified by peer review) is the author/funder. This article is a US Government work. It is not subject to copyright under 17 USC 105 and is also made available for use under a CC0 license.

Supplementary Figure 2

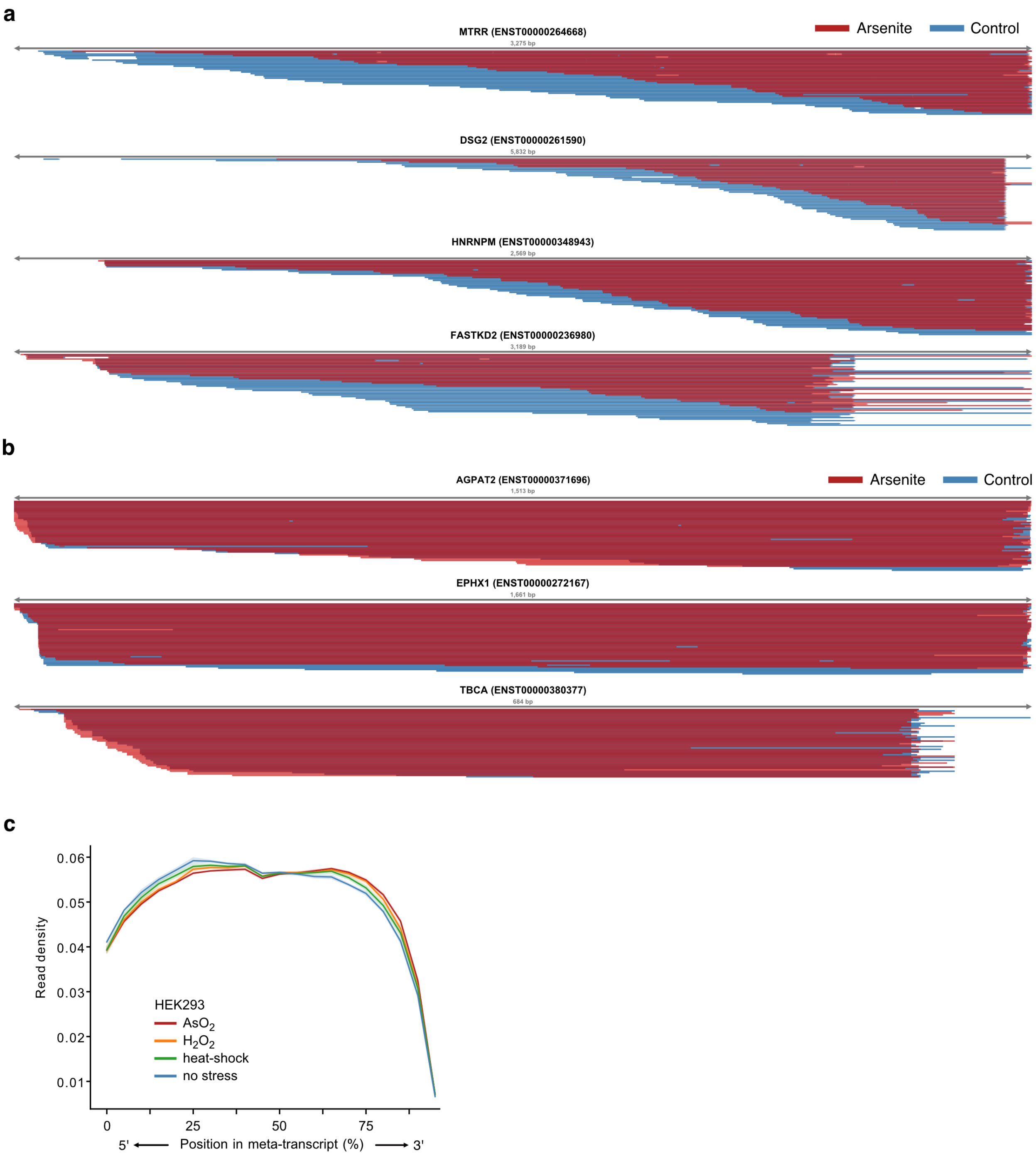

bioRxiv preprint doi: https://doi.org/10.1101/2023.08.31.555629; this version posted September 1, 2023. The copyright holder for this preprint (which was not certified by peer review) is the author/funder. This article is a US Government work. It is not subject to copyright under 17 USC 105 and is also made available for use under a CC0 license.

Supplementary Figure 3

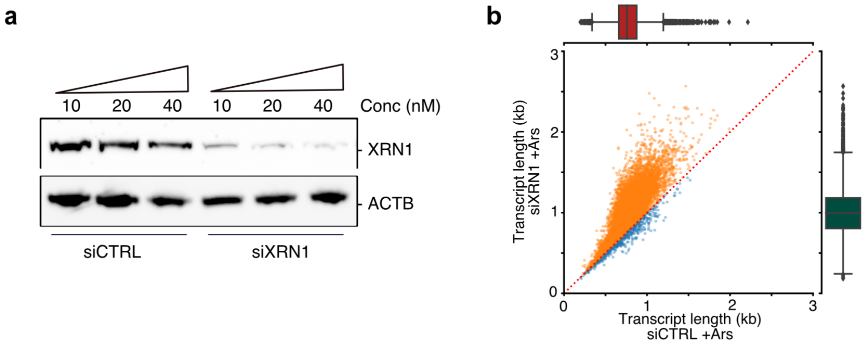

bioRxiv preprint doi: https://doi.org/10.1101/2023.08.31.555629; this version posted September 1, 2023. The copyright holder for this preprint (which was not certified by peer review) is the author/funder. This article is a US Government work. It is not subject to copyright under 17 USC 105 and is also made available for use under a CC0 license.

Supplementary Figure 4

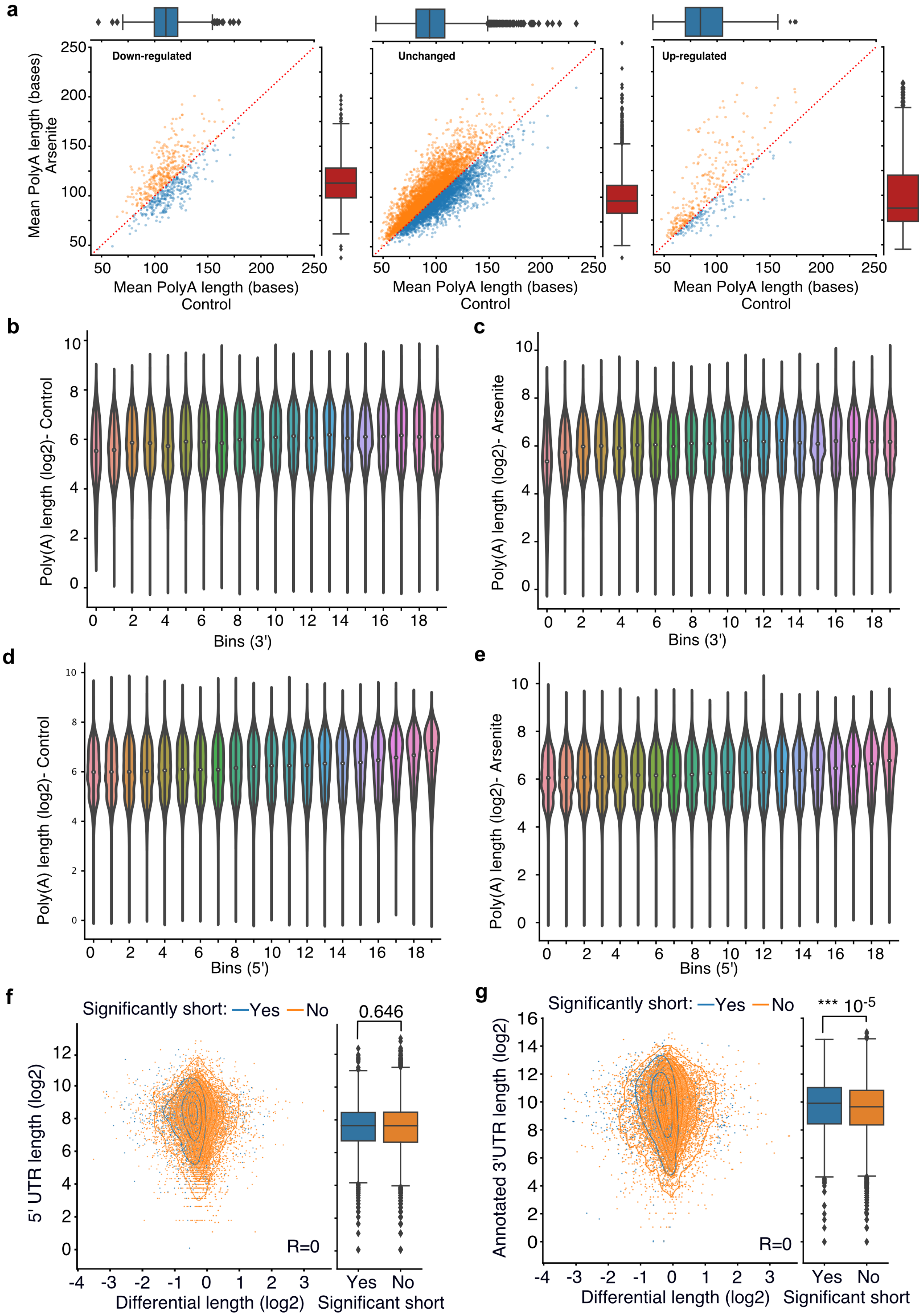

bioRxiv preprint doi: https://doi.org/10.1101/2023.08.31.555629; this version posted September 1, 2023. The copyright holder for this preprint (which was not certified by peer review) is the author/funder. This article is a US Government work. It is not subject to copyright under 17 USC 105 and is also made available for use under a CC0 license.

Supplementary Figure 5

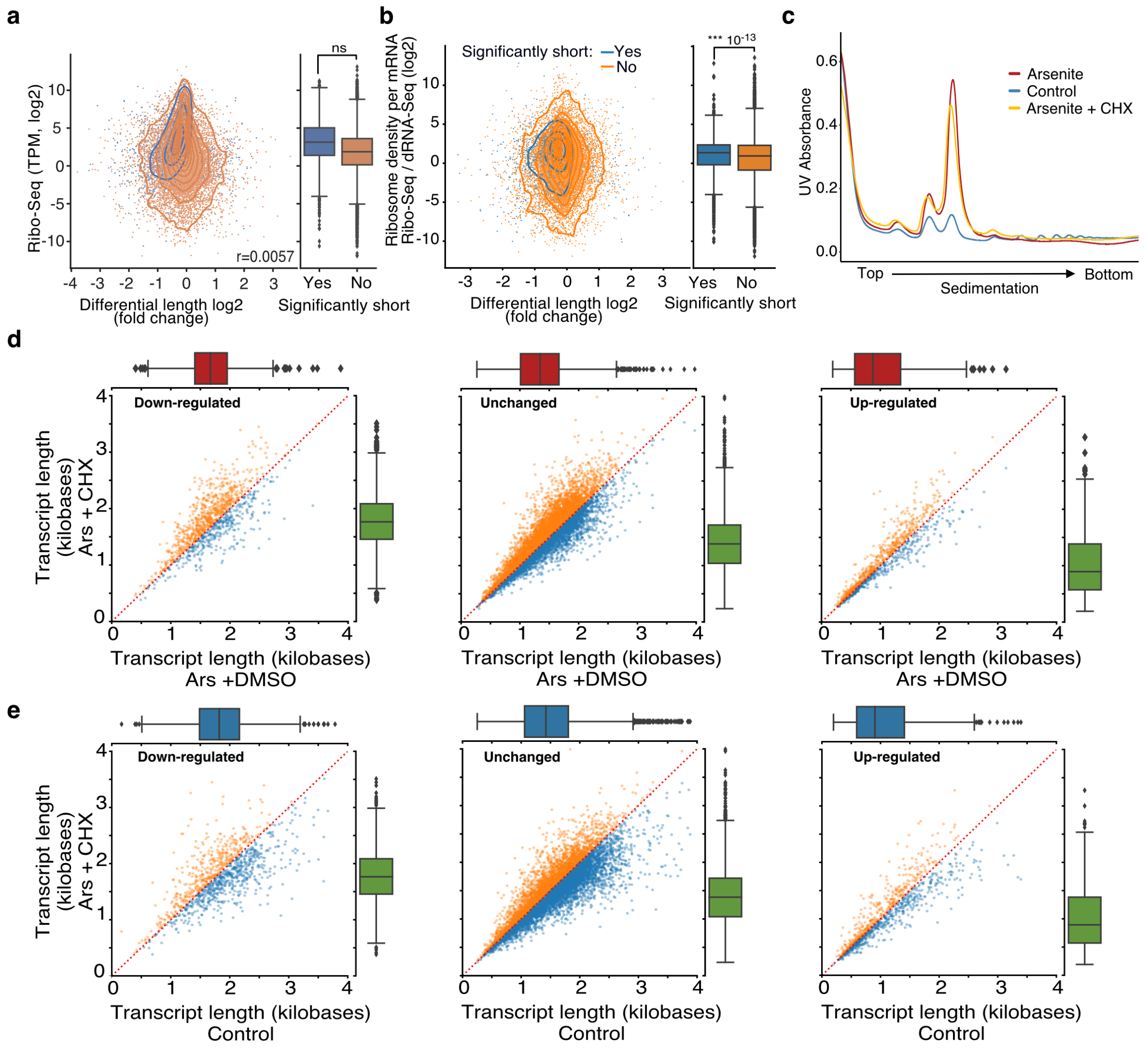

bioRxiv preprint doi: https://doi.org/10.1101/2023.08.31.555629; this version posted September 1, 2023. The copyright holder for this preprint (which was not certified by peer review) is the author/funder. This article is a US Government work. It is not subject to copyright under 17 USC 105 and is also made available for use under a CC0 license.

Supplementary Figure 6

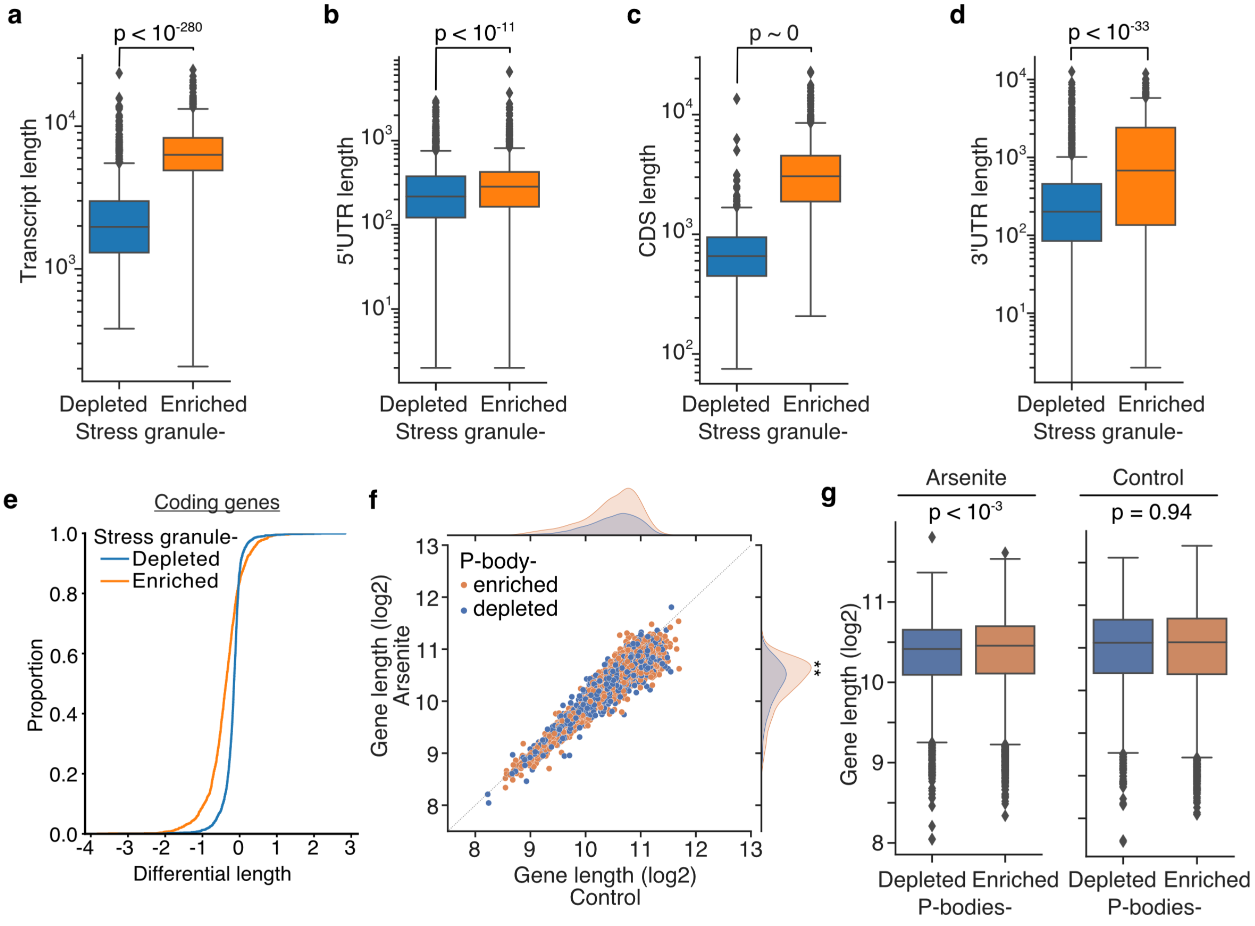

Supplement: Supplementary file 1 [file NIHPP2023.08.31.555629v1-supplement-5.pdf]
